# Memory B-cells elicited by different HPV vaccine regimens in the DoRIS randomised controlled trial

**DOI:** 10.1038/s41541-025-01313-8

**Published:** 2025-11-28

**Authors:** Rebecca Wiggins, Kathy J. Baisley, Jackton Indangasi, John Changalucha, Helen Ashwin, Najmeeyah Brown, Hilary S. Whitworth, David Joakim, Philippe Mayaud, Ramadhan Hashim, Caroline Maxwell, Paul Mutani, Beatrice Kamala, Brett Lowe, Ligia Pinto, Troy Kemp, Silvia deSanjosé, Saidi Kapiga, Richard J. Hayes, Deborah Watson-Jones, Charles J. Lacey

**Affiliations:** 1 York Biomedical Research Institute, Hull York Medical School, University of York, York, UK; 2 London School of Hygiene and Tropical Medicine, Keppel Street, London, UK; 3 Mwanza Intervention Trials Unit, National Institute for Medical Research, Mwanza, Tanzania; 4 Vaccine, Immunity and Cancer Directorate, Frederick National Laboratory for Cancer Research, Frederick, MD USA; 5 Barcelona Institute for Global Health (ISGlobal), Barcelona, Spain

**Keywords:** Immunology, Diseases, Health care, Medical research

## Abstract

Prophylactic human papillomavirus vaccines such as Cervarix® and Gardasil®9 induce robust and sustained antibody responses over time. One driver of such responses is memory B-cells (MBCs), primed during initial HPV-vaccine exposure. As part of the Dose Reduction Immunobridging and Safety Study (DoRIS), MBCs were evaluated in peripheral blood mononuclear cells by enzyme-linked immunosorbent spot assay at baseline and at 5 visits post-first vaccine dose to Month 36 in 930 Tanzanian girls randomly allocated to 3, 2, or 1 doses of either vaccine. Most ( > 90%) participants had detectable MBCs at all time points, with maximum responses by Month 7. Geometric mean frequency HPV-specific MBCs and the proportion responding declined thereafter for both vaccines in the 2 and 3-dose arms. MBC frequencies were lower in single-dose than in 2- and 3-doses recipients at all time points subsequent to Month 1. By Month 36, MBC responses to both vaccines for both HPV16 and 18 were similar across all dosing arms. The clinical significance of the dose response remains to be evaluated; however, the presence of MBCs in the circulation after 3 years with a single vaccine dose is encouraging in terms of generating long-lasting protection with a one-dose vaccination strategy.

## Introduction

Cervical cancer is the fourth most common cancer among women globally, and the most common cancer in women in many sub-Saharan African countries, with 90% of new cases and deaths occurring in low- and middle-income countries (LMICs)^1,2^. The widespread introduction of vaccination programmes against oncogenic human papillomavirus (HPV) genotypes 16 and 18 and other high-risk types has led to reductions in the incidence of cervical cancer and pre-cancer in vaccinated populations^3,4^. For LMICs, implementation of such vaccination programmes has been predicted to reduce the incidence of cervical disease by ~90% within the next century, even without cervical screening^5^. Currently available vaccines include the 2-valent HPV16/18 *Cervarix®* (GlaxoSmithKline Biologicals), the 4-valent HPV-6/11/16/18 *Gardasil®* (Merck and Co., Inc.), the 9-valent HPV-6/11/16/18/31/33/45/52/58 *Gardasil®9* (Merck and Co., Inc.), the 2-valent 16/18 vaccines *Cecolin™*^6^ and *Walrinvax™*^7^ in China, and the 4-valent 6/11/16/18 *Cervavac*^*TM*^ in India^8^. All these vaccines are L1 protein virus-like particle (VLP) vaccines and induce robust protection against HPV vaccine-genotype infection^9,10^.

In 2022, the World Health Organization (WHO) recommended that a 2-dose schedule be used for individuals from 9 years of age, and that a single-dose schedule could be used as an off-label option in girls and boys aged 9–20 years^11^, based on strong evidence of efficacy in comparisons against placebo in post-hoc analyses from two large studies, one prospective randomised controlled trial, and immunobridging studies^12–15^. Reducing the number of doses would increase access to vaccination and decrease the costs of routine programme implementation^16,17^.

The leading immunological correlates of protection from HPV disease are not entirely clear, although experimental evidence indicates neutralizing antibodies are the key mechanism^18,19^. Long-term antibody production depends on efficient immunological priming driving the generation of long-lived plasma cells (LLPCs) and memory B-cell responses^20^. Whereas LLPCs reside in the bone marrow, which makes direct measurement of these cells challenging, memory B-cells are a circulating lymphocyte subset that give rise to plasmablasts and plasma cells and may be a surrogate for effective priming of long-lived antibody responses. Memory B-cells are primed during initial HPV vaccine exposure^21^. B-cell division occurs when the cells are first exposed to antigen and then make the fate choice to differentiate into either memory B-cells or specific IgG-producing plasma cells. Repeated antigen-specific exposure amplifies the immune response^20,22^ Protection against infection and disease, and maintenance of anti-HPV VLP antibodies, can persist for up to 14 years with the three dose regimen^4,10^, and is predicted to extend up to 20 years^23^. HPV VLP antigen-specific memory B-cell responses are present for at least 6 years post-vaccination^24,25^.

The Dose Reduction Immunobridging and Safety Study (DoRIS)^14,26^ was designed with the following co-primary objectives: 1. to demonstrate non-inferiority of immune responses following a single dose of Cervarix® or Gardasil®9 compared with two and three doses of the same vaccine, measured by seropositivity to HPV 16 and 18 in a VLP enzyme-linked immunosorbent assay (ELISA), in a population of Tanzanian girls aged between 9 and 14 years; 2. to demonstrate non-inferiority of antibody geometric mean concentrations (GMC) of vaccine-specific antibodies at Month 24, when comparing the 1 dose regimen of the vaccines with historical cohorts of women aged 10–25 years who received one dose, in whom efficacy has been demonstrated^14,15,26^ In order to address the underlying mechanisms of immune responses to HPV vaccination and investigate their persistence, a secondary objective was to examine the maintenance and magnitude of vaccine-specific memory B-cell responses in the same study population, comparing single dose Cervarix® or Gardasil®9, to two and three doses, at months 1, 7, 12, 24 and 36 post-vaccination.

## Results

### Baseline characteristics of the cohort and numbers of peripheral blood samples available for analysis

Nine hundred and thirty healthy girls aged between 9 and 14 years from Mwanza, Tanzania were enrolled into the DoRIS trial and randomly assigned to receive one, two or three doses of either Cervarix® or Gardasil®9 (155 per arm). Eighteen of the 930 (1.9%) girls recruited into the study self-reported as having passed sexual debut^14^. At baseline, 57 girls (6%) were seropositive for HPV16 and 81 (9%) seropositive for HPV18^14^ (Table 1). The 930 participants contributed 5522 peripheral blood mononuclear cell (PBMC) samples across the 6 study visits (Supplementary Table 1). Of the 5522 samples available across all timepoints, 5341 (96.7%) were included in the final analysis for the TVC. A small proportion (55, or <0.5%) had low viable cell counts ( <75%) on thaw and were excluded from the analysis. Despite viability >75%, a further 126 samples had low total IgG ( < 100 total IgG positive spots in the first dilution (50,000 PBMCs)) following stimulation and were also excluded from the analysis. This could occur even when viability and cell count on thaw fell within the necessary parameters ( >0.9 × 10^5^; >80% viability) to take the cells forward to the stimulation culture.

**Table 1:** Demographic characteristics and HPV positivity at baseline in the DoRIS trial. Data are median (IQR) or n (%)

	1 dose 2-valent (*n* = 155)	2 doses 2-valent (*n* = 155)	3 doses 2-valent (*n* = 155)	1 dose 9-valent (*n* = 155)	2 doses 9-valent (*n* = 155)	3 doses 9-valent (*n* = 155)	Total (*n* = 930)
Age (years)	10 (9–12)	11 (10–12)	10 (9–12)	10 (9–12)	11 (10–13)	11 (9–13)	10 (9–12)
Age group							
9–10 years	85 (54·8%)	74 (47·7%)	85 (54·8%)	88 (56·8%)	70 (45·2%)	73 (47·1%)	475 (51·1%)
11–12 years	39 (25·2%)	45 (29·0%)	36 (23·2%)	41 (26·5%)	45 (29·0%)	41 (26·5%)	247 (26·6%)
13–14 years	31 (20·0%)	36 (23·2%)	34 (21·9%)	26 (16·8%)	40 (25·8%)	41 (26·5%)	208 (22·4%)
HPV 16 DNA positive	0	0	1 (0·6%)	1 (0·6%)	0	1 (0·6%)	3 (0·3%)
HPV 18 DNA positive	0	0	2 (1·3%)	1 (0·6%)	0	0	3 (0·3%)
High-risk HPV genotype DNA	0	2 (1·3%)	4 (2·6%)	6 (3·9%)	2 (1·3%)	3 (1·9%)	17 (1·8%)
HPV genotype DNA	0	2 (1·3%)	4 (2·6%)	7 (4·5%)	2 (1·3%)	5 (3·2%)	20 (2·2%)

### Proportion of subjects with detectable antigen-specific memory B-cells in 1, 2 and 3-dose arms

**Fig. 1:**
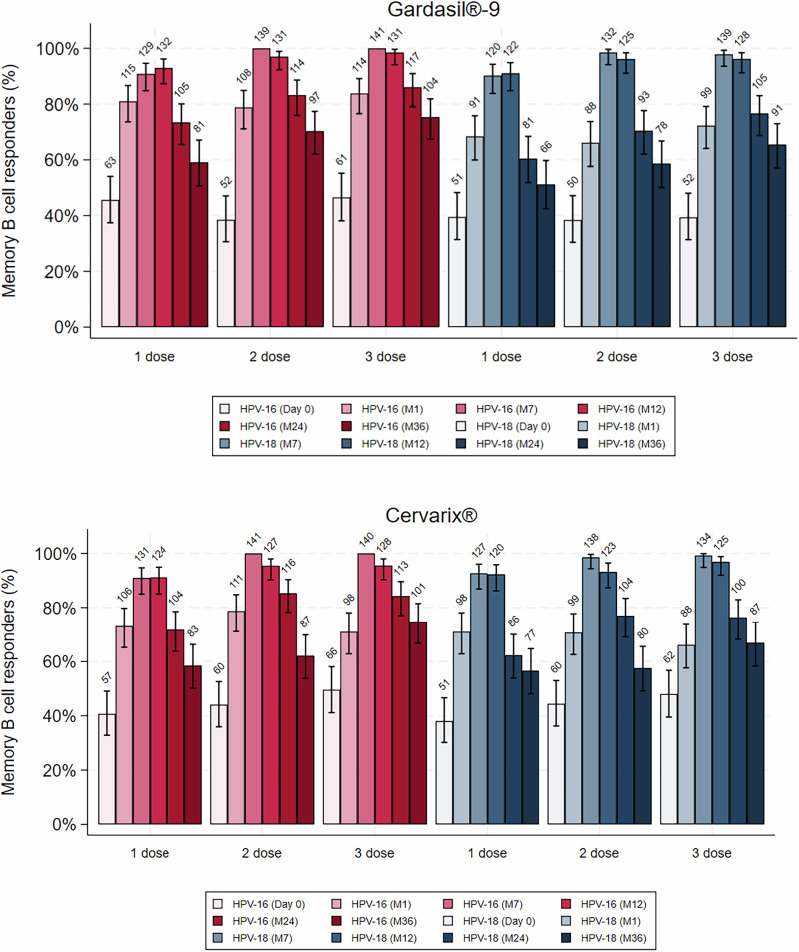
Proportion with detectable HPV-specific B-cell responses over time (per protocol cohort). Bars represent 95% CI, and numbers are the number of participants with detectable responses.

The proportion of subjects with detectable HPV16 and 18-specific memory B-cells ( > 0 antigen-specific memory B-cell/1 × 10^5^ total IgG-producing cells, expressed as percentages), geometric means (GM) of the circulating HPV-specific memory B-cells, and 95% confidence intervals (CI) in the PP cohort are shown in Fig. 1 and Supplementary Table 2. At entry (Day 0), before HPV vaccination, approximately 40% of girls had detectable HPV 16 and 18 VLP memory B-cells, although these responses were of very low magnitude (discussed below).

At Month 1, after girls in all arms had received the first dose of vaccine, the mean proportion of responders (participants who had a demonstrable memory B-cell response) across the arms in the PP cohort ranged from 71.0% to 78.7% (Cervarix®, HPV16), 66.2–71.0% (Cervarix®, HPV18), 78.8–83.8% (Gardasil®9, HPV16), and 66.2–72.3% (Gardasil®9, HPV18) (Supplementary Table 2). There was approximately a five-fold increase in geometric mean memory B-cell frequency at Month 1 in those positive for memory B-cells at Day 0 (Supplementary Table 2).

In the 1 dose arms, the peak proportions of responders were seen at 7 and 12 months (i.e., 7 and 12 months post-single dose). In the 2- and 3-dose groups the peak proportion of responders was seen at 7 months (i.e., 1-month post-last dose, for either the two or three dose regimens), with a marginal drop at Month 12. Responses were almost complete at Month 7 in the 2 and 3-dose arms (98.5– 100%), in contrast to the single dose arms (90.2–92.7%), Supplementary Table 2). The same pattern was also seen in terms of the magnitude of the response, discussed below. At 12 months, response rates were above 90% across all dosing arms [91.2–95.5% (Cervarix®, HPV16), 92.3–96.9% (Cervarix®, HPV18), 93.0 – 98.5% (Gardasil®9, HPV16), and 91.0–96.2% (Gardasil®9, HPV18), Supplementary Table 2). There was a progressive decline in the proportion of responders to both vaccines from Month 12 to Month 36 across all dose groups.

### Frequencies of antigen-specific memory B-cells in 1, 2 and 3-dose arms

**Fig. 2:**
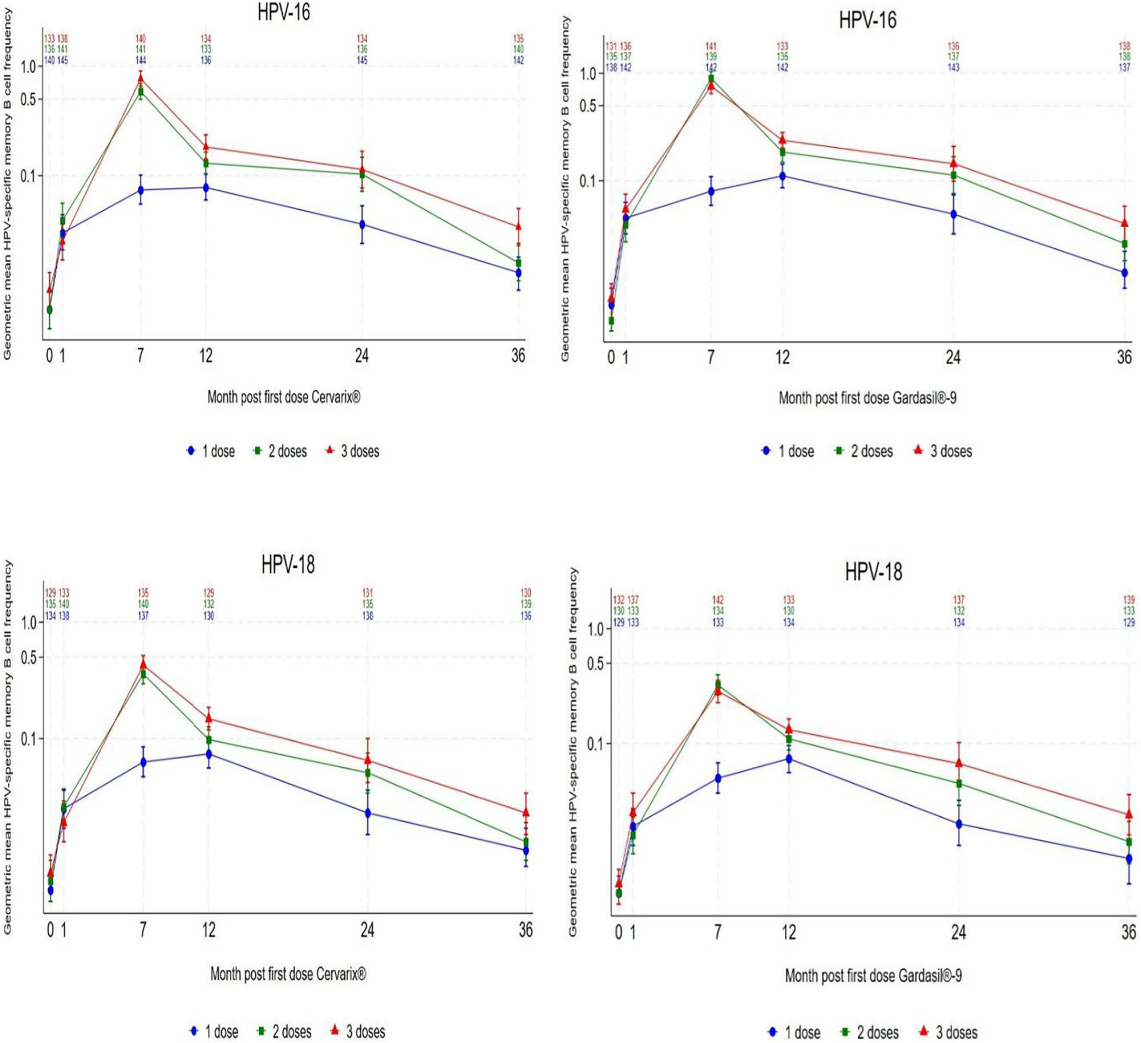
Trajectory of geometric means for HPV-specific memory B-cells by study visit and number of doses of Cervarix® and Gardasil®9(per-protocol cohort). Top panels, HPV16-specific memory B-cells;® bottom panels; HPV18-specific memory B-cells. Panels for Cervarix®, left; for Gardasil®9, right. Numbers in each cohort are displayed above each dose/timepoint.

**Fig. 3:**
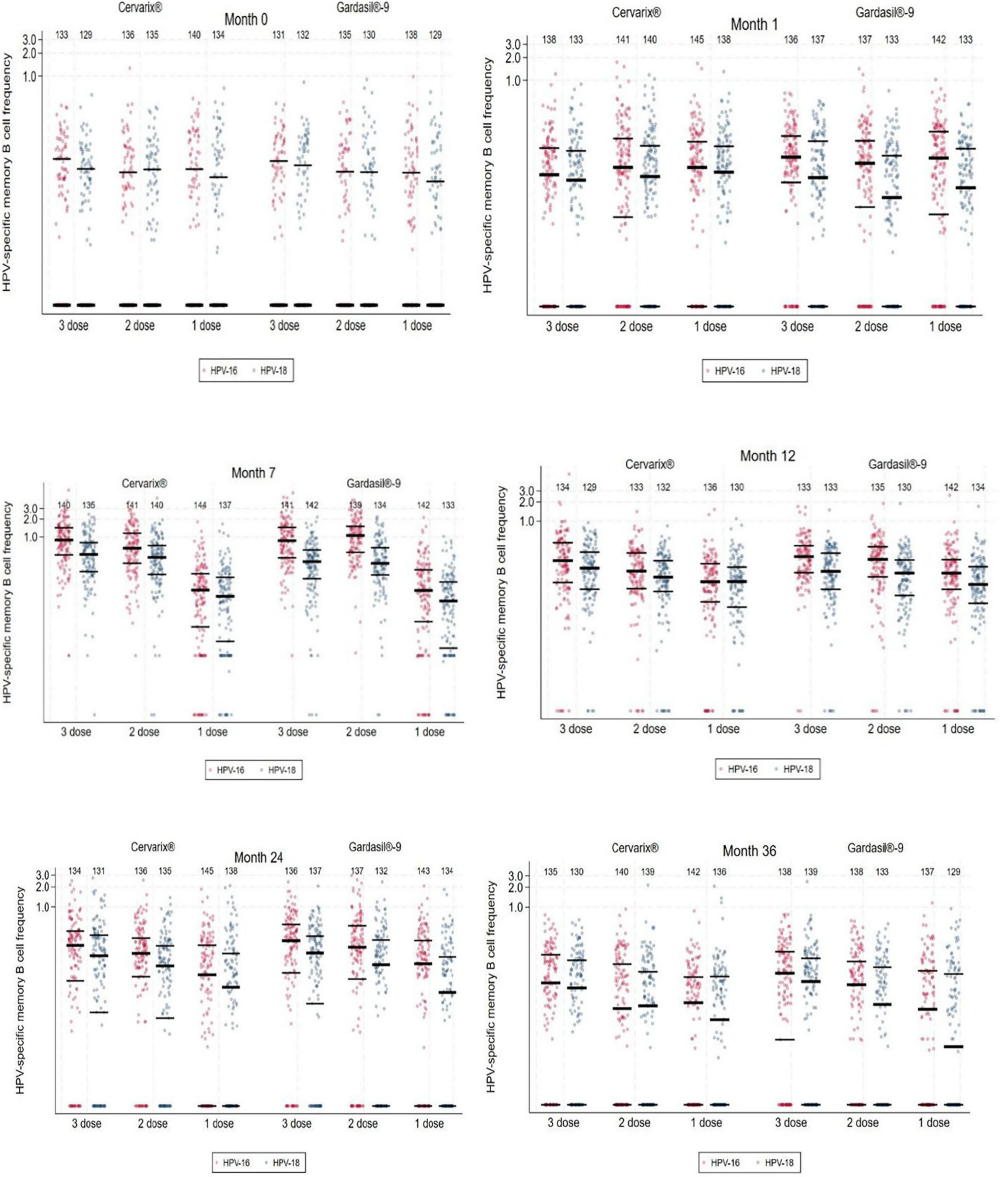
Frequency of HPV-specific memory B-cell responses (percentage of HPV-specific circulating memory B-cells of total IgG-producing cells) demonstrating the overlapping distribution at months 1, 7, 12, 24, and 36, by arm (per protocol cohort) (median and IQR). Each data point represents a single individual.

Frequencies of memory B-cells, defined as the percentage of antigen-specific memory B-cell/1 × 10^5^ total IgG producing cells) for the PP cohort are shown in Figs. 2 and 3, and in Supplementary Table 2. In terms of detectable antigen-specific memory B-cells, we observed the following differences in the responses between the 1 dose arms, and the 2- and 3-dose arms, for both Cervarix® and Gardasil®9, as described below. In the 2 and 3-dose arms, the highest frequencies of detectable antigen-specific memory B-cells were seen at Month 7 for both vaccines and both HPV 16 and 18; these frequencies were 6- to 11-fold higher in the 2- and 3-dose arms than in the 1-dose arms. There was considerable overlap in memory B-cell frequencies between the dose groups at all timepoints, particularly at Month 36 (Fig. 3 and Supplementary Table 2).

Figure 2 illustrates the kinetics of the responses over time. Over the first 12 months of follow-up, the 1 dose arms show similar peak GM frequencies of HPV-specific memory B-cells at Month 7 (Cervarix® HPV16 GM 0.074, 95% CI: 0.055–0.101; HPV18 GM 0.063, 95% CI: 0.047–0.085; Gardasil®9 HPV16 GM 0.080, 95% CI: 0.059–0.109; HPV18 GM 0.050, 95% CI: 0.037–0.068, Supplementary Table 2) and at Month 12 (Cervarix® HPV16 GM 0.078, 95% CI: 0.06–0.103; HPV18 0.074, 95% CI: 0.056–0.096; Gardasil®9 HPV16 GM 0.111, 95% CI: 0.086–0.143; HPV18 GM 0.074, 95% CI: 0.056–0.096, Supplementary Table 2) whereas the frequency of HPV-specific memory B-cells in the 2 and 3-dose arms was highest at Month 7 (e.g. Cervarix® HPV16 3-doses GM 0.769, 95% CI: 0.655–0.903: Gardasil®9 HPV18 3-doses GM 0.285, 95% CI: 0.227–0.356, Supplementary Table 2) and displayed a steep decline by Month 12 (Cervarix® HPV16 3-doses GM 0.183, 95% CI: 0.142–0.236; Gardasil®9 HPV18 GM 0.132, 95% CI: 0.106–0.164 (Supplementary Table 2). The trajectory of the single-dose arms differed between Month 1 and Month 12 in comparison to the 2 and 3-dose arms, which had a pronounced peak at Month 7, shortly following the Month 6 vaccination, and then a fall at Month 12. These patterns are similar to those seen in the overall responder rates. From Month 12 to Month 36 there was a decline in HPV16 and HPV18 specific memory B-cell frequencies across all arms (Fig. 2).

### Comparison of GM memory B-cell frequencies and ratios between dose groups

For both vaccines, overall geometric mean (GM) HPV16 and HPV18 memory B-cell frequencies from Month 7 to Month 36 were higher in the 3-dose arms than 2-dose arms, although the difference was not statistically significant except for 3-dose Cervarix® at Month 12 (HPV18) (95% CI: 0.47–0.94) and Month 36 (HPV16) (95% CI: 0.28–0.82) (Table 2). There was a significant difference in HPV-specific GM memory B-cell ratios between 1 and 3-doses of both vaccines at all timepoints (Table 2). Geometric mean memory B-cell frequencies were significantly lower in the 1 dose arms than the 2-dose arms from Month 7 to Month 24. At Month 36, there was no evidence of a difference in HPV-specific GM memory B-cell frequencies between the 1 and 2-dose arms for Cervarix® for HPV16 or HPV18, or for Gardasil®9 for HPV18, and the difference was only of borderline significance for HPV16 for Gardasil®9 (95%CI = 0.32–0.96, Table 2). However, given the wide confidence intervals and the reduced number of positive spots at Month 36 in comparison to other timepoints from Months 7 to 24, statistical inferences may be of limited value. These patterns were reproduced in the TVC (Supplementary Data Table 2).

**Table 2:** Comparisons of HPV-specific geometric mean (GM) memory B cell concentrations at M12 after 1, 2 or 3 doses of HPV vaccine in DoRIS trial (per-protocol cohort^1^)

		1 dose		2 doses		3 doses	Geometric mean memory B cell ratio (95% CI)		
	N^2^	GM^3^ (95% CI)	N^2^	GM^3^ (95% CI)	N^2^	GM^3^ (95% CI)	1 dose / 2 dose	1 dose / 3 dose	2 dose / 3 dose
					Month 1 Cervarix®				
HPV-16	145	0.030 (0.021–0.044)	141	0.039 (0.027–0.056)	138	0.025 (0.017–0.036)	0.78 (0.47–1.29)	1.22 (0.73–2.04)	1.58 (0.94–2.64)
HPV-18	138	0.025 (0.017–0.036)	140	0.026 (0.018–0.037)	133	0.019 (0.013–0.029)	0.97 (0.57–1.64)	1.27 (0.75–2.18)	1.32 (0.77–2.25)
					Gardasil-9®				
HPV-16	142	0.045 (0.032 -0.063)	137	0.039 (0.027–0.055)	136	0.054 (0.039–0.075)	1.15 (0.72–1.86)	0.83 (0.51–1.33)	0.72 (0.44–1.16)
HPV-18	133	0.019 (0.013–0.027)	133	0.016 (0.011–0.023)	137	0.025 (0.018–0.037)	1.15 (0.68–1.94)	0.73 (0.44–1.23)	0.64 (0.38–1.07)
					Month 7 Cervarix®				
HPV-16	144	0.074 (0.055–0.101)	141	0.588 (0.498–0.694)	140	0.769 (0.655–0.903)	0.13 (0.09–0.17)	0.10 (0.07–0.13)	0.76 (0.56–1.04)
HPV-18	137	0.063 (0.047–0.085)	140	0.359 (0.295–0.438)	135	0.428 (0.356–0.516)	0.18 (0.13–0.24)	0.15 (0.11–0.20)	0.84 (0.61–1.16)
					Gardasil-9®				
HPV-16	142	0.080 (0.059–0.109)	139	0.897 (0.774–1.039)	141	0.758 (0.648–0.886)	0.09 (0.07–0.12)	0.11 (0.08–0.14)	1.18 (0.87–1.61)
HPV-18	133	0.050 (0.037–0.068)	134	0.324 (0.265–0.397)	142	0.285 (0.227–0.356)	0.15 (0.11–0.22)	0.18 (0.12–0.25)	1.14 (0.81–1.61)
					Month 12 Cervarix®				
HPV-16	136	0.078 (0.060–0.103)	133	0.130 (0.104–0.164)	134	0.183 (0.142–0.236)	0.60 (0.42–0.86)	0.43 (0.30–0.61)	0.71 (0.50–1.02)
HPV-18	130	0.074 (0.056–0.096)	132	0.098 (0.076–0.126)	129	0.148 (0.119–0.185)	0.75 (0.53–1.06)	0.50 (0.35–0.70)	0.66 (0.47–0.94)
					Gardasil-9®				
HPV-16	142	0.111 (0.086–0.143)	135	0.185 (0.149–0.231)	133	0.238 (0.201–0.281)	0.60 (0.44–0.81)	0.47 (0.34 –0.64)	0.78 (0.57–1.06)
HPV-18	134	0.074 (0.056–0.096)	130	0.110 (0.088–0.138)	133	0.132 (0.106–0.164)	0.67 (0.48–0.94)	0.56 (0.40–0.78)	0.84 (0.60–1.17)
					Month 24 Cervarix®				
HPV-16	145	0.036 (0.024–0.053)	136	0.103 (0.072–0.147)	134	0.114 (0.077–0.167)	0.35 (0.20–0.59)	0.31 (0.18–0.54)	0.91 (0.53–1.56)
HPV-18	138	0.023 (0.015–0.036)	135	0.051 (0.034–0.075)	131	0.065 (0.042–0.100)	0.45 (0.25–0.82)	0.35 (0.20–0.64)	0.78 (0.43–1.42)
					Gardasil-9®				
HPV-16	143	0.049 (0.032–0.074)	137	0.113 (0.076–0.167)	136	0.144 (0.099–0.209)	0.43 (0.25–0.75)	0.34 (0.20–0.59)	0.78 (0.45–1.37)
HPV-18	134	0.020 (0.013–0.032)	132	0.045 (0.029–0.070)	137	0.067 (0.044–0.102)	0.45 (0.25–0.84)	0.30 (0.17–0.56)	0.67 (0.36–1.23)
					Month 36 Cervarix®				
HPV-16	142	0.013 (0.009–0.018)	140	0.016 (0.011–0.024)	135	0.034 (0.023–0.050)	0.78 (0.46–1.32)	0.37 (0.22–0.63)	0.48 (0.28–0.82)
HPV-18	136	0.011 (0.008–0.017)	139	0.013 (0.009–0.019)	130	0.023 (0.015–0.034)	0.86 (0.50–1.50)	0.50 (0.28–0.87)	0.58 (0.33–1.01)
					Gardasil-9®				
HPV-16	137	0.014 (0.010–0.022)	138	0.026 (0.018–0.038)	138	0.040 (0.027–0.058)	0.55 (0.32–0.96)	0.36 (0.21–0.63)	0.66 (0.38–1.13)
HPV-18	129	0.010 (0.006–0.014)	133	0.014 (0.009–0.021)	139	0.024 (0.016–0.036)	0.68 (0.38–1.21)	0.40 (0.23–0.71)	0.59 (0.33–1.03)

^1^DoRIS participants who were ELISA antibody negative & DNA negative at baseline (pre-vaccination) for the HPV genotype under analysis. ^2^Number with detectable HPV-specific memory B cells at Month 36. ^3^Geometric mean % of HPV-specific circulating memory B cells. Values below the assay limit of quantitation (LLQ) are set to 0.5*LLQ for analysis.

### Participants who had pre-existing anti-IgG responses at baseline

Between 38.1% (51/134, Cervarix®, HPV18) and 49.6% (66/133, Cervarix®, HPV16) of girls in the PP cohort of the DoRIS trial had evidence of low-level HPV16/18-specific -specific memory B-cell responses at baseline (GM 0.005 – 0.009), with no evidence of a significant difference between arms (p ≥ 0.48). As a sensitivity analysis, we excluded these participants from the analysis of the proportion of responders and HPV-specific GM memory-B cell frequencies, to see if the results differed from those based on the full PP cohort (Supplementary Table 4 Supplementary Figs. 6-8). The trajectory of the single dose arms between Month 7 and Month 12 in the Gardasil®9 arms for both genotypes was slightly flatter when those who had memory B-cells at baseline were excluded, but there was no marked difference between the groups when those who had memory B-cells at baseline were either included or excluded (Fig. 2, Supplementary Fig. 7).

### Association between Month 7 memory B-cells and anti-HPV IgG antibody

**Fig. 4:**
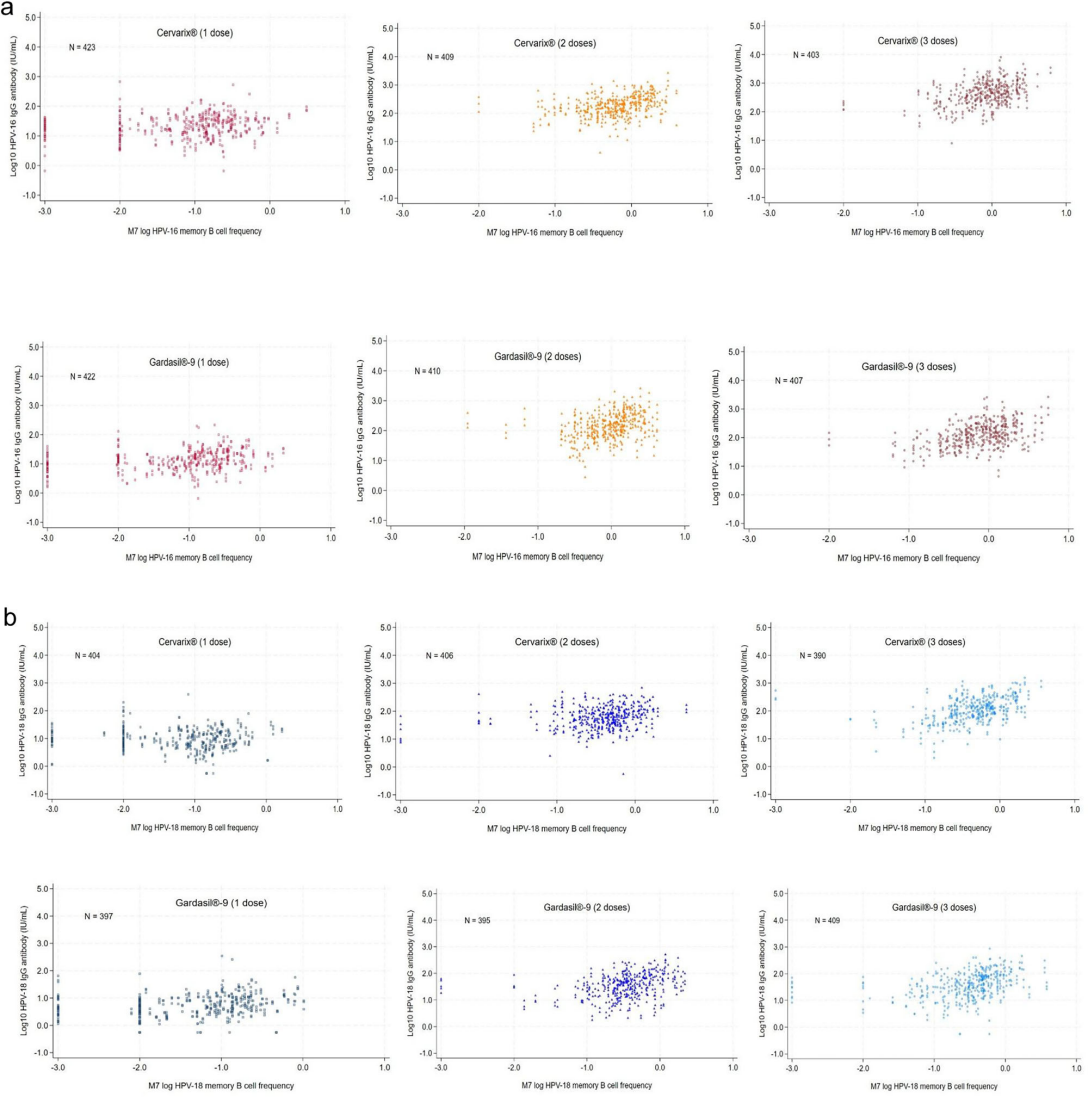
Trajectory of geometric means for HPV-specific memory B-cells for Cervarix® and Gardasil®9 (per-protocol cohort). Top panels, HPV16 memory B-cells; bottom panels, HPV18 memory B-cells. Panels for Cervarix®, left; for Gardasil®9, right. **a** Month 7 log % HPV16-specific memory B-cells against log10 anti-HPV IgG antibody at Month 12 through Month 36. Each participant contributes multiple observations to the scatterplot. **b** Month 7 log % HPV18-specific memory B-cells against log10 anti-HPV IgG antibody at Month 12 through Month 36. Each participant contributes multiple observations to the scatterplot.

In the 2 and 3-dose arms, there was strong evidence of a positive association of Month 7 memory B-cells frequencies with anti-HPV IgG antibody concentrations from Month 7 to Month 36, for both vaccines and both HPV genotypes (Table 3 and Fig. 4a, b), with HPV antibody concentrations increasing with increasing memory B-cell frequencies. The magnitude of the association was generally greater (a larger increase) for 3-doses than 2-doses, except for HPV18 for Gardasil®9, where 2 and 3-doses were similar.

**Table 3:** Association between log memory B cell responses at M7 and anti-HPV IgG concentrations after 1, 2 and 3-doses (per-protocol cohort^1^)

	1 dose			2-doses			3-doses		
Visit	N	Fold increase (95% CI)^2^	p-value^3^	N	Fold increase (95% CI)^2^	p-value^2^	N	Fold increase (95% CI)^2^	p-value^2^
Cervarix®									
HPV-16									
M7	144	1.45 (1.22–1.72)	<0.001	141	1.87 (1.36–2.57)	<0.001	140	2.69 (1.93–3.74)	<0.001
M12	136	1.37 (1.12–1.66)	0.002	133	2.05 (1.41–2.98)	<0.001	134	3.46 (2.31–5.17)	<0.001
M24	145	1.20 (1.00–1.42)	0.044	136	1.90 (1.37–2.63)	<0.001	134	2.29 (1.64–3.21)	<0.001
M36	142	1.21 (1.02–1.44)	0.026	140	1.93 (1.42–2.62)	<0.001	135	2.46 (1.79–3.38)	<0.001
HPV-18									
M7	137	1.04 (0.85–1.26)	0.707	140	1.37 (1.04–1.81)	0.027	135	2.23 (1.63–3.04)	<0.001
M12	130	0.90 (0.73–1.13)	0.370	132	1.40 (1.03–1.89)	0.031	129	2.60 (1.84–3.68)	<0.001
M24	138	0.97 (0.79–1.19)	0.783	135	1.54 (1.15–2.05)	0.003	131	2.41 (1.75–3.31)	<0.001
M36	136	0.88 (0.72–1.09)	0.240	139	1.43 (1.08–1.91)	0.013	130	2.46 (1.77–3.42)	<0.001
Gardasil-9®									
HPV-16									
M7	142	1.17 (0.98–1.40)	0.086	139	1.82 (1.24–2.67)	0.002	141	1.97 (1.38–2.81)	<0.001
M12	142	1.29 (1.09–1.51)	0.003	135	1.89 (1.34–2.68)	<0.001	133	2.90 (2.09–4.01)	<0.001
M24	143	1.33 (1.11–1.58)	0.002	137	2.15 (1.49–3.12)	<0.001	136	2.71 (1.92–3.82)	<0.001
M36	137	1.28 (1.07–1.52)	0.006	138	2.18 (1.50–3.16)	<0.001	138	2.69 (1.91–3.79)	<0.001
HPV-18									
M7	133	1.23 (1.02–1.50)	0.034	134	1.57 (1.17–2.09)	0.002	142	1.55 (1.21–1.98)	0.001
M12	134	1.24 (1.01–1.52)	0.037	130	1.81 (1.34–2.44)	<0.001	133	1.89 (1.45–2.46)	<0.001
M24	134	1.38 (1.11–1.72)	0.004	132	1.80 (1.30–2.50)	<0.001	137	1.87 (1.41–2.49)	<0.001
M36	129	1.35 (1.08–1.68)	0.008	133	1.94 (1.35–2.77)	<0.001	139	1.97 (1.49–2.60)	<0.001

^1^DoRIS participants who were ELISA antibody negative & DNA negative at baseline (pre-vaccination) for the HPV genotype under analysis. ^2^Increase in mean log anti-HPV IgG concentrations (IU/mL) with 1 unit increase in M7 log HPV-specific memory B cells. ^3^*p*-value from linear regression at each visit, with log IgG concentrations as the response and fixed effects for M7 log HPV-specific memory B cells, dose and their interaction.

In the 1 dose arms, there was also evidence of a positive association of Month 7 memory B-cells responses with HPV antibody concentrations from Month 7 to Month 36 for both HPV genotypes for Gardasil®9, and for HPV16 but not HPV18 for Cervarix®. In the 1 dose arm of Cervarix®, a 1-unit increase in Month 7 log-transformed HPV16-specific memory B-cells was associated with a 1.21-fold increase in geometric mean HPV16 antibody concentrations at Month 36 (95% C I = 1.02 – 1.44) (Table 3). In the 1 dose Gardasil®9 arm, a 1-unit increase in Month 7 log HPV16 memory B-cells was associated with a 1.28-fold (95% CI = 1.07 – 1.52) increase in GM HPV16 antibody concentrations at Month 36.

## Discussion

Trials of mulitdose schedules of Cervarix® and Gardasil®9 have shown high efficacy associated with enduring seropositivity. The current recommended regimen is for 2 doses with 1 dose allowed as an alternative regimen. Trials have also shown high efficacy of the single dose regimen^12,15,27,28^. To explore the breadth of the immune response to these vaccines over time, we assessed the magnitude and durability of memory B-cell responses to three and two doses of HPV vaccine, compared to a single dose, over 36 months among Tanzanian girls aged between 9 and 14 years, as a secondary objective of the DoRIS trial^14^. Frequencies of memory B-cells as a percentage of total IgG and the number of responders in the 6 arms were compared. We also examined the association between the memory B-cell response at Month 7 and HPV16 and 18 antibody seropositivity at all visits to Month 36 post-first dose. To our knowledge, this is the first study from sub-Saharan Africa to analyse memory B-cell responses to a single dose of HPV vaccine.

These results demonstrate that memory B-cells are induced after the first dose of both vaccines, peak in the 2 and 3-dose arms at Month 7, and are detectable in the circulation in all three dose groups up to Month 36 (the last visit in this analysis). There was a stepwise increase in mean memory B-cell frequencies with each additional dose for both vaccines and for both HPV genotypes at Months 7 to 36. The memory B-cell responses in the 2- and 3-dose arms were not significantly different at almost all timepoints, in keeping with other studies of memory B-cell responses to HPV vaccines^29,30^ apart from Cervarix® 2-doses compared to 3-doses at Month 12 (for HPV18) and Month 36 (for HPV16). In the Gardasil®9 arms the peak frequency was slightly, but not significantly, higher in the 2-dose arm at Month 7 for HPV16 than in the 3-dose arm. Memory B-cell frequencies in the single dose arms from Month 7 to Month 36 were lower than 2- and 3-dose arms. By Month 36 there was no significant difference between 1- and 2-dose groups in HPV-specific GM memory B-cell frequencies, except for HPV16 in the 1-dose Gardasil®9 arm. These results, in terms of both number of responders and memory B-cell frequency from ELISpots, are similar to other studies that examined long term follow-up of HPV vaccine-induced cellular and serological responses^24,25,31–34^, although true like-to-like comparisons are limited by methodological variations. The results in the TVC (which includes subjects who were HPV DNA/seropositive at baseline) were similar to those in the PP cohort. Detection of low-level baseline memory B-cell responses pre-vaccination at Day 0 is in keeping with other studies^25,30^, and may be explained by some form of exposure in childhood to HPV vaccine-related HPV genotypes^35,36^. It is also possible that sexual exposure had occurred in some cases, and that sexual activity was under-reported.

The single dose arms for both vaccines and both genotypes initially followed a different (shallower) trajectory compared to the 2 and 3-dose arms. In the single dose arms, there was an increase in memory B-cell frequency from Month 1 to Month 7 and then again to Month 12, although the increase between Month 7 and Month 12 was not statistically significant (Fig. 2). In contrast, there was a marked increase from Month 1 to Month 7 in the 2 and 3-dose arms and then a significant decline in these arms at Month 12. Thereafter memory B-cell frequencies declined progressively in all dose groups until Month 36, although a majority of participants still exhibited a response with both vaccines and both genotypes at Month 36 in all arms. This contrasted with IgG antibody responses from the same samples in all dose groups, which remained stable across the timepoints, including in a 1 dose and 2 dose comparison^14,32^. Antibody stability is probably due to the structure of the HPV VLPs in subunit vaccines, which induce potent activation signals to naïve B cells leading to the consistent induction of LLPCs which maintain antibody levels, although this mechanism may not explain why the memory B-cell levels in the one-dose arm at Month 12 are similar to those of the one-dose arm at Month 7. ^9,37^. Exactly how long antigen-presenting follicular dendritic cells retain antigens such as HPV VLPs in lymph nodes and thus promote induction of B-cell^9^, including memory B-cell, responses is unclear, although evidence suggests this may be for up to 12 months, or even longer^38^, which may have an influence on the durability of the 1-dose memory B-cells. Caution must however be applied in the interpretation of our data because lower frequencies of memory B-cells, more commonly seen in later timepoints and single dose arms, are closer to the limit of assay detection, and the likelihood of false positives or false negatives is increased. However, the number of 1-dose memory B-cells at Month 12 in this study appear similar to those noted in an earlier study^25^ which analysed responses to reduced dose schedules of Cervarix® at 12 months.

One study noted a lower HPV16-specific memory B-cell recall response at Month 7 in subjects given the standard 3-dose regimen of quadrivalent Gardasil® in those individuals who already had a high serum antibody titre at the time of the third dose. In contrast, the magnitude of the response was then greater in these participants after a subsequent boost at Month 24. The authors postulated that the timing of the multidose HPV vaccine schedule may therefore be critical to ensuring an optimal memory B-cell response^39^. In malaria vaccine trials, subjects with an enforced delayed boosting regimen had enhanced responses for both antigen-specific memory B-cells and LLPCs^40^. Our data may indicate that boosting at M6 may not make a substantial difference to the long-term maintenance of anti-HPV antibody responses, as memory B-cells are detected in 91–93% of single dose subjects at Month 12, but subsequently display similar decay kinetics to 2- and 3-dose subjects.

The geometric mean frequencies of HPV16 and 18-specific memory B-cells and the number of responders to both genotypes were generally higher for HPV16 than 18 in the majority of timepoints, which is consistent with most of the serology results in the published literature^41–46^ However, overall the proportion of responders was similar between the two vaccines. A previous study observed that the magnitude of memory B-cell responses were largely equivalent for Gardasil®4 and Cervarix®^24^. We observed that the frequency of detectable memory B-cells was higher overall in Gardasil®9 compared to Cervarix® vaccinees for HPV16 at all timepoints, although this difference was less apparent by Month 36. One study noted it may be more difficult to draw firm conclusions from later timepoints regarding memory B-cell measurements, as in the absence of challenge many memory B-cells may have taken up tissue residence^31^ and are thus undetectable in the circulation.

We were able to test for an association between memory B-cell frequency at Month 7 and anti-HPV serum antibodies, as had been previously performed in other studies^14,24,26^. Similarly to a study which examined the potential correlation between Month 7 memory B-cells and antibodies to HPV16^47^, we found a significant association between memory B-cell frequency and anti-HPV antibody concentrations at Month 7 and subsequent time points to Month 36, with 2 and 3-doses of either vaccine. There was also a significant association between Month 7 HPV16-specific memory B-cell frequency and anti-HPV16 antibodies from Month 7 to Month 36 after a single dose of either vaccine; however, there was no evidence of an association between Month 7 HPV18-specific memory B-cell frequency and anti-HPV18 antibodies from Month 7 to Month 36 with Cervarix® after a single dose of HPV vaccine. Another study found there was an association 6 months post the third dose, but not after at least 2 years, between memory B-cells and HPV antibody levels after a 3-dose vaccination series^24^. Memory B-cells are not always closely correlated with the presence of serum antibodies after vaccination or natural infection. Two principal theories have been proposed for this observation; that long-lived plasma cells maintain antibody levels independently^37^, or that plasma cells require replenishment by the proliferation and differentiation of memory B-cells^21,24,48^. Antibody levels have been shown to be maintained following some infections or vaccinations over 26 years, with memory B-cells associated with antibody levels for measles, mumps and rubella, but not for vaccinia, Epstein-Barr Virus, Varicella Zoster Virus, tetanus or diphtheria^38^. Recent data suggest that upon secondary challenge (e.g. sexual exposure to HPV) circulating memory B-cells may enter the female reproductive tract and act as the source of rapid and robust local luminal antibody secretion^49^.

A principal advantage of this study is the comprehensive analysis of memory B-cells across the first 3 years of follow-up from the first randomised trial of the 1 dose schedule in the primary target age range for HPV vaccination, with a detailed description of memory B-cell kinetics. Our study also provides the first data on memory B cell responses from sub-Saharan Africa. Interestingly, unlike HPV-specific antibody levels, which essentially plateau after 12 months across 2 or 3-doses of either HPV vaccine, with this plateau able to be maintained out to > 10 years^4,10,50^, memory B-cells responders and geometric mean frequencies of memory B-cells are still gradually declining out to 36 months after the initial or sole vaccine dose. Seminal studies of smallpox and other viral vaccines and infections have demonstrated that plateau levels of memory B-cells are indeed reached after 5–10 years and they then may persist out to ~ 50 years^38,51^. Sixty-month follow-up data from the DoRIS trial demonstrates ongoing stability of HPV 16 and HPV 18 IgG antibody concentrations following 1 and 2-doses of either HPV vaccine^32^. Similarly, follow-up studies of the bivalent and quadrivalent three, two and one-dose comparisons reported persistent HPV16 and HPV18 seropositivity among women who received a single dose of these vaccines up to 11 (bivalent vaccine) and 9 (quadrivalent vaccine) years earlier^52,53^. Our data support these findings and may be encouraging in terms of durable vaccine-induced immunity to HPV, and the role of memory B-cells for long-term priming, although extended surveillance is required to ascertain if this does apply with the HPV vaccines, particularly with single-dose administration and/or particular patient groups such as those living with human immunodeficiency virus^54^. A recent study demonstrated that HPV-specific memory B-cells have been detected 10 years post-vaccination in subjects who had previously received 2 or 3-doses of HPV vaccine and were given a booster dose of Gardasil®9 dose at 10 years, indicating HPV-specific memory B-cells are still present and able to be recalled on challenge^55^. The KEN SHE trial, which examines B cell-responses at 36 and 37 months in the context of the efficacy of single-dose vaccination, is another example of a long-term follow-up study which includes evaluation of immune cell memory in its study design^56^.

The strengths of our study include a large sample size with an excellent retention rate and multiple timepoint sampling to Month 36, with a robust experimental design including optimal sample processing, shipping and storage. Preparation of the cells for analysis was undertaken using well-established standard operating procedures and a validated ELISpot technique (Mabtech B-cell ELISpot^57,58^).

This study has limitations. There was no assessment in the Gardasil®9 arms of the other targeted HPV- vaccine types (6, 11, 31, 33, 45, 52). The trial also focussed on the main target group for vaccination, namely girls from 9–14 years, and did not incude a study of male vaccination and subsequent immune responses^59,60^, although there is currently a trial of the single dose schedule being undertaken in Tanzania with boys aged 14–18 years (Add-Vacc, NCT04953130).

Each timepoint had the same number of cells plated in each well, e.g. wells for Month 1 and Month 36 samples both contained 100,000 cells for the antigen-specific coated wells. For the latter months, particularly Month 36, there may be a loss of sensitivity, as the study demonstrates that memory B cell frequency fell from Months 24 to Month 36, therefore a higher number of cells in each well may have increased the likelihood of detecting HPV-specific memory B-cells at later timepoints. It is possible that some of the Month 36 samples fell below the limit of detection, particularly in the single dose groups, and that samples which did have antigen-specific memory B-cells fell below the detection limit simply because the number of HPV-specific memory B-cells were of very low abundance. Our approach was however consistent across timepoints and was the same as that of similar studies^25,31^.

The high level of manipulation of cellular samples required for the memory B-cell ELISpot carries a risk of suboptimal performance of the cells during the assay. Polyclonal stimulation is necessary in order to induce in vitro differentiation into antibody-producing plasma cells^61^. We considered this risk to be minimized due to the slightly shorter activator incubation time (72 h as opposed to 5 – 6 days) recommended with the mitogens used (IL-2 and R848).

Antigen-specific memory B-cell spots were present at very low levels in 30–50% of subjects at Day 0, prior to vaccination, with no evidence of a difference between the trial arms. Subjects who were either HPV16 or 18 DNA seropositive were included in the TVC. The memory B-cell spots present in the day zero samples may be due to cross-reactivity with other common non-oncogenic HPV types, or the subjects may have had previous HPV16/18 infection but had cleared this by the time of recruitment, although given the young age of the participants this is less likely. There was some limited crossover of HPV-specific memory B-cell frequencies with values at other timepoints, particularly Month 36.

In conclusion, the DoRIS HPV vaccination trial provided the opportunity to examine the breadth of immune reponses to two extensively employed HPV vaccines across various immunological parameters, and to expand the analysis of immune cell memory into reduced-dose regimens in the target age group for vaccination. There was broad agreement with other studies in regard to the frequency and durability of memory B-cells with vaccination. The strong correlation of memory B-cell frequency at Month 7 and antibody responses across dose arms to Month 36 indicates there may be priming of the immunological response at an early stage for the production of antibodies at later timepoints, thus providing further evidence of the robust, durable, and comprehensive immune reponses to these highly efficacious vaccines^62^. The demonstration that HPV-specific memory B-cells, although reduced in number compared to earlier timepoints, can be found in the circulation three years after a single dose of vaccine is encouraging with regard to potential long-lasting protection against oncogenic HPV genotypes following a single dose of HPV vaccine. Further research is required to confirm durable protection following single-dose HPV vaccination.

## Methods

### *Study population* (Clinicaltrials.gov registration NCT02834637)

Nine hundred and thirty healthy HIV-negative girls aged 9–14 years in Mwanza city, Tanzania, who had no prior HPV vaccination and no chronic or underlying immune conditions were randomised to 1 of 6 arms (155 per arm) comprising 3 different dose schedules of the 2-valent vaccine [Cervarix®, GSK Biologicals, Rixensart] or the 9-valent vaccine [Gardasil®9], Sanofi Pasteur MSD, Lyon]: 3-doses over 6 months, 2-doses given 6 months apart, or a single dose. Written or fingerprinted informed consent was obtained from parents or guardians, and assent from participants. The per protocol (PP) cohort was defined as DoRIS participants who were ELISA antibody negative and DNA negative at baseline (pre-vaccination) for the HPV genotype under analysis. The total vaccinated cohort (TVC) was defined as DoRIS participants who received at least one dose of vaccine, irrespective of their HPV serostatus or DNA status at baseline^26^.

### Peripheral blood mononuclear cells (PBMCs)

Whole blood ( ~ 10 mL) was drawn into sodium heparin vacutainers (BD, Berkshire, UK). Blood separation for isolation of peripheral blood mononuclear cells (PBMCs) was performed in a microbiological class II safety cabinet (MSC) at the National Institute for Medical Research (NIMR) laboratory in Mwanza, using 12 mL Leucosep™ tubes (Greiner Bio-One, Glos, UK) within 6 h of collection, with R10 (RPMI 1640 (Sigma-Aldrich, Dorset, UK) 10% FBS (Hyclone, ThermoFisher, Loughborough, UK) with the addition of 1% penicillin-streptomycin (Gibco, Thermo Fisher, UK) and 2 mM L-glutamine (Sigma-Aldrich) as the extraction media (DoRIS NIMR laboratory standard operating procedure (SOP) T076, available on request). Cells were counted using a Countess II automated counter according to NIMR laboratory SOP T077, aliquoted in freezing media (9 mL heat-inactivated fetal bovine serum (FBS) and 1 ml dimethyl sulphoxide (DMSO, Merck) and frozen according to NIMR laboratory SOP T064 at a concentration of ~1 × 10^7^.

Isolated PBMC samples were removed from −150 °C storage and transported to University of York, UK in liquid nitrogen (LN_2_) Cryo-Express (dry) shippers. Samples were cross-checked and stored in Human Tissue designated LN_2_ storage dewars until thawing for memory B-cell analysis. Available samples at each timepoint are shown in Table 1 (Results).

In preparation for memory B-cell ELISpots, samples were removed from LN_2_, thawed rapidly in a 37 °C water bath and extracted into 9 mL R10. Samples were centrifuged to remove freezing media and resuspended in 1 mL R10 for counting. During counting, cells were rested for a minimum of 1 hour with the lids of the tubes loosened in a CO_2_ incubator (5% CO_2_). Counts were conducted using a Vi-Cell XR automated counter (Beckman Coulter, High Wycombe, UK) with parameters specific to PBMC identification with trypan blue exclusion to assess viability. A manual alternative counting system (Kova® slide, Fisher Scientific, Loughborough, UK) was used according to manufacturers’ instructions if the Vi-Cell was unavailable.

### Polyclonal stimulation of PBMCs

After counting and prior to stimulation, cells from each participant were diluted to 1.5 × 10^6^/mL in R10. In vitro stimulation is required to promote differentiation of memory B-cells cells into antibody-secreting cells. The final volume for stimulation (1.2 mL) was aliquoted into Cellstar® Cellreactor tubes (Fisher Scientific). The TLR7/8 agonist R848 (resiquimod) @1μg/mL and IL-2 @ 10 ng/mL (both Mabtech, Sweden) for B-cell proliferation were added to the tubes and incubated for ~72 h at 37 °C in 5% CO_2_. Cell suspensions were then removed from the incubator, centrifuged to remove the stimulation media and resuspended in 1 mL R10. A repeat cell count was performed. Cells were diluted to 1 × 10^6^ for application to the prepared ELISpot plate.

There was a < 2% loss of samples on thaw, and across all timepoints the average number of samples rejected from the final analysis due to low IgG post-activation was 3.3%. Initial cell viability data from the trial site demonstrated a low inter-operator variability and a highly efficient PBMC isolation procedure.

### ELISpots

The ELISpot plate preparation was as follows: MAIPS4510, or if unavailable MSIPS 96 well PVDF Immobilon membrane plates (Merck-Millipore, Dorset, UK) were pre-wetted with 35% (MAIPS) or 70% (MSIPS) ethanol and washed in sterile dH_2_O. For each participant sample, positive control wells were coated with 100μL anti-human IgG (MT91/145 (3850-2H, Mabtech, Nacka Strand, Sweden) @ 15 μg/mL in PBS + 0.5% FBS). Negative control wells were coated with either 100 μL keyhole limpet hemocyanin (KLH, Merck) @ 20 μg/mL or 100 μL anti-human IgG for media-only wells. Triplicate antigen-specific wells were coated with either 100μL HPV 16 or 18 virus-like particles (VLPs) (donated by Glaxo SmithKline, Belgium) @10μg/mL. Plates were incubated overnight – 5 days @ 2–8 °C, then washed in sterile PBS and blocked either for 1 h at RT or overnight @ 2–8 °C with 200 μL R10. After tapping off the R10, doubling dilutions of stimulated cells with a starting concentration of 0.5 × 10^5^cells/well were applied to positive control wells. All other wells (antigen-specific and KLH-coated) received 1 × 10^5^ cells apart from media-only wells. In addition, samples from known HPV 16 and 18 vaccinated recruits (AMBER study: REC reference 17/YH/0333, unpublished data, including HPV16/18 negative PBMC samples from volunteers recruited by convenience sampling) and HPV16/18 negative PBMC samples were run as additional positive and negative controls. After the addition of analyte, plates were incubated @ 37 °C for 16–24 h in CO_2_ incubator.

The cell suspension was removed by washing on an automated plate washer (Skanwasher 300) 4x in PBS prior to addition of 100 μL biotinylated anti-IgG antibody (MT78/145 @1 μg/mL, Mabtech) for 2 h at room temperature. Plates were washed again in PBS, incubated with 100 μL streptavidin-HRP in the dark for 1 h, followed by a further wash and addition of filtered 100 μL tetramethyl benzidine (TMB) precipitating substrate for ELISpot (Mabtech). Plates were developed for 12 min, washed in deionized water and left to dry upside down overnight in the dark prior to counting on an AID Classic enzymatic reader (Oxford Biosystems). After counting the dried plates were stacked and kept in the dark to prevent fading.

Wells containing spots that were deemed too many to count were not included in the total IgG analysis; instead, the well with the next concentration in the serial dilution was used to represent the IgG count^58^, after subtraction of spots in negative control wells. Samples with sparse total IgG spots (<100 in the 50,000 cell well) were eliminated from the final analysis. Memory B-cells were identified as the number of IgG-secreting cells in anti-human IgG-coated wells. Antigen-specific memory B-cells were defined as detectable spots in VLP 16 and 18 coated wells above the number of detectable spots in negative control wells. Antigen-specific spots tend to be larger and more diffuse when not biotinylated compared to total IgG spots^58^. Numbers in the total IgG wells were adjusted to represent 1 × 10^5^ cells and the results presented as antigen-specific Memory B-cells /1 × 10^5^ cells. Results were expressed as frequency or percentage of antigen-specific spots/total IgG spots and each spot represented 1 IgG-secreting cell. Positive wells were defined as >0 antigen-specific memory B-cell/1 × 10^5^ total IgG producing cells, expressed as percentages, after subtraction of spots in media controls or irrelevant antigen controls. Operators were blinded to arm, but not timepoint, during experimentation and ELISpot counting.

Samples were processed in batches to reduce variation and fully vaccinated and unvaccinated samples were used to establish assay parameters and were run periodically with the DoRIS samples to monitor assay performance. Antibody lots and mitogens were tested on positive control samples to check consistency between lots as this was a long-running trial and the same lots of reagents could not be used in all cases due to expiry dates. A secondary negative control in addition to a media control was run with each sample to increase the robustness of the assay. Operators were blinded to arm, but not timepoint.

Some samples were excluded from the final analysis due to poor total IgG reponses following mitogen stimulation. This could occur even when viability and cell count on thaw fell within the necessary parameters ( > 0.9 × 10^5^; >80% viability) to take the cells forward to the stimulation culture. Ideally, all of these samples would be retested; however approximately one third did not have an additional sample vial available to thaw, and the cost of shipping duplicate samples from Tanzania was prohibitive. Duplicate samples from Month 24 (*n* = 26) which had stimulated poorly were however included in a scheduled shipment and tested. As there was an inadequate response for these duplicate samples also, this may indicate a functional problem inherent in the samples themselves, rather than the stimulation and/or assay not performing optimally on these occasions.

### Statistical analysis

Analyses were performed on the PP cohort and exploratory analyses on the TVC. Memory B-cell frequencies expressed as percentage antigen-specific/total IgG spots were log_10_ transformed for analysis. Geometric mean (GM) HPV-specific memory B-cells frequencies and 95% confidence intervals (CI) were obtained by back-transformation. The difference in log10 memory B-cell frequencies between dose groups within the same vaccine (e.g., 1 dose vs 3-doses of the 2-valent vaccine) was calculated at each time point with its 95% CI; the GM ratio and its 95% CI were obtained by back-transformation. Statistical significance was defined as a 2-sided *p*-value < 0.05.

The association between HPV-specific memory B-cell frequencies at Month 7 and IgG anti-HPV VLP concentrations over time was analysed using a mixed-effects linear regression model. The response variable was the log10-transformed anti-HPV IgG antibody concentrations at months 7, 12, 24, and 36, and the independent variables were Month 7 log10- transformed memory B-cell frequencies, visit, dose group and visit-dose group interaction, with a random effect for participant to account for correlation of repeated measurements within participant. Separate models were run for each vaccine and HPV genotype. SAS (version 9.1) and Stata (version 18) were used for all analyses.

## Supplementary information

Wiggins et al. memory B-cell DoRIS Supplementary Data

## Data Availability

De-identified participant data presented in this manuscript can be made available after publication following written request to the London School of Hygiene & Tropical Medicine and the Mwanza Intervention Trials Unit, Tanzania. Requests must be accompanied by a defined analysis plan addressed to the corresponding author which will be reviewed by the Mwanza Intervention Trials Unit Data Sharing Committee and senior investigators at the London School of Hygiene & Tropical Medicine. Requesting researchers will be required to sign a Data Access Agreement if approval is given.

## References

[1] Hull, R. et al. Cervical cancer in low and middle-income countries. *Oncol. Lett.* **20**, 2058–2074 (2020). 32782524 10.3892/ol.2020.11754 PMC7400218

[2] Sung, H. et al. Global Cancer Statistics 2020: GLOBOCAN Estimates of Incidence and Mortality Worldwide for 36 Cancers in 185 Countries. *CA Cancer J. Clin.* **71**, 209–249 (2021). 33538338 10.3322/caac.21660

[3] Palmer, T. et al. Prevalence of cervical disease at age 20 after immunisation with bivalent HPV vaccine at age 12-13 in Scotland: retrospective population study. *BMJ* **365**, l1161 (2019). 30944092 10.1136/bmj.l1161 PMC6446188

[4] Kjaer, S. K. et al. Final analysis of a 14-year long-term follow-up study of the effectiveness and immunogenicity of the quadrivalent human papillomavirus vaccine in women from four nordic countries. *EClinicalMedicine* **23**, 100401 (2020). 32637895 10.1016/j.eclinm.2020.100401 PMC7329692

[5] Brisson, M. et al. Impact of HPV vaccination and cervical screening on cervical cancer elimination: a comparative modelling analysis in 78 low-income and lower-middle-income countries. *Lancet* **395**, 575–590 (2020). 32007141 10.1016/S0140-6736(20)30068-4 PMC7043009

[6] Zou, Z. et al. Domestic HPV vaccine price and economic returns for cervical cancer prevention in China: a cost-effectiveness analysis. *Lancet Glob. Health* **8**, e1335–e1344 (2020). 32971056 10.1016/S2214-109X(20)30277-1

[7] You, T. et al. Optimal allocation strategies for HPV vaccination introduction and expansion in China accommodated to different supply and dose schedule scenarios: A modelling study. *EClinicalMedicine* **56**, 101789 (2023). 36618898 10.1016/j.eclinm.2022.101789 PMC9813696

[8] Burki, T. K. India rolls out HPV vaccination. *Lancet Oncol.* **24**, e147 (2023). 36934729 10.1016/S1470-2045(23)00118-3

[9] Schiller, J. & Lowy, D. Explanations for the high potency of HPV prophylactic vaccines. *Vaccine* **36**, 4768–4773 (2018). 29325819 10.1016/j.vaccine.2017.12.079 PMC6035892

[10] Artemchuk, H. et al. Long-term Antibody Response to Human Papillomavirus Vaccines: Up to 12 Years of Follow-up in the Finnish Maternity Cohort. *J. Infect. Dis.* **219**, 582–589 (2019). 30239832 10.1093/infdis/jiy545

[11] Organization, W. H. Human papillomavirus vaccines: WHO position paper. *Wkly Epidemiological Rec.* **50**, 645–672 (2022).

[12] Barnabas, R. V. et al. Durability of single-dose HPV vaccination in young Kenyan women: randomized controlled trial 3-year results. *Nat. Med* **29**, 3224–3232 (2023). 38049621 10.1038/s41591-023-02658-0 PMC10719107

[13] Whitworth, H. S. et al. Efficacy and immunogenicity of a single dose of human papillomavirus vaccine compared to no vaccination or standard three and two-dose vaccination regimens: A systematic review of evidence from clinical trials. *Vaccine* **38**, 1302–1314 (2020). 31870572 10.1016/j.vaccine.2019.12.017

[14] Watson-Jones, D. et al. Immunogenicity and safety of one-dose human papillomavirus vaccine compared with two or three doses in Tanzanian girls (DoRIS): an open-label, randomised, non-inferiority trial. *Lancet Glob. Health* **10**, e1473–e1484 (2022). 36113531 10.1016/S2214-109X(22)00309-6 PMC9638030

[15] Baisley, K. et al. Comparing one dose of HPV vaccine in girls aged 9-14 years in Tanzania (DoRIS) with one dose of HPV vaccine in historical cohorts: an immunobridging analysis of a randomised controlled trial. *Lancet Glob. Health* **10**, e1485–e1493 (2022). 36113532 10.1016/S2214-109X(22)00306-0 PMC9638025

[16] Benard, E. et al. Potential population-level effectiveness of one-dose HPV vaccination in low-income and middle-income countries: a mathematical modelling analysis. *Lancet Public Health* **8**, e788–e799 (2023). 37777288 10.1016/S2468-2667(23)00180-9 PMC10557953

[17] Trimble, C. L. & Trimble, E. L. HPV vaccines: when one plus one equals three. *Lancet Glob. Health* **10**, e1373–e1374 (2022). 36113514 10.1016/S2214-109X(22)00373-4

[18] Pinto, L. A. et al. Immunogenicity of HPV prophylactic vaccines: Serology assays and their use in HPV vaccine evaluation and development. *Vaccine* **36**, 4792–4799 (2018). 29361344 10.1016/j.vaccine.2017.11.089 PMC6050153

[19] Roy, V. et al. Differences in HPV-specific antibody Fc-effector functions following Gardasil(R) and Cervarix(R) vaccination. *NPJ Vaccines* **8**, 39 (2023). 36922512 10.1038/s41541-023-00628-8 PMC10017795

[20] Palm, A. E. & Henry, C. Remembrance of Things Past: Long-Term B Cell Memory After Infection and Vaccination. *Front Immunol.* **10**, 1787 (2019). 31417562 10.3389/fimmu.2019.01787 PMC6685390

[21] Prabhu, P. R., Carter, J. J. & Galloway, D.A. B. Cell Responses upon Human Papillomavirus (HPV) Infection and Vaccination. *Vaccines.* **10**, 837 (2022). 35746445 10.3390/vaccines10060837 PMC9229470

[22] Siegrist, C.-A. Vaccine immunology. *In Vaccines* 6th edn (eds Plotkin, S. A., Orenstein, W. A. & Offit, P. A.) ch. 2, 17–36 (Elsevier, 2012).

[23] Schwarz, T. et al. Persistence of immune responses to the HPV-16/18 AS04-adjuvanted vaccine in women aged 15-55 years and first-time modelling of antibody responses in mature women: results from an open-label 6-year follow-up study. *BJOG* **122**, 107–118 (2015). 25208608 10.1111/1471-0528.13070 PMC4489326

[24] Nicoli, F. et al. HPV-Specific Systemic Antibody Responses and Memory B Cells are Independently Maintained up to 6 Years and in a Vaccine-Specific Manner Following Immunization with Cervarix and Gardasil in Adolescent and Young Adult Women in Vaccination Programs in Italy. *Vaccines (Basel).* **8**, 26 (2020). 31947611 10.3390/vaccines8010026 PMC7175219

[25] Pasmans, H. et al. Long-term HPV-specific immune response after one versus two and three doses of bivalent HPV vaccination in Dutch girls. *Vaccine* **37**, 7280–7288 (2019). 31575492 10.1016/j.vaccine.2019.09.066

[26] Baisley, K. J. et al. A dose-reduction HPV vaccine immunobridging trial of two HPV vaccines among adolescent girls in Tanzania (the DoRIS trial) - Study protocol for a randomised controlled trial. *Contemp. Clin. Trials* **101**, 106266 (2021). 33421649 10.1016/j.cct.2021.106266 PMC7970022

[27] Kreimer, A. R. et al. Efficacy of a bivalent HPV 16/18 vaccine against anal HPV 16/18 infection among young women: a nested analysis within the Costa Rica Vaccine Trial. *Lancet Oncol.* **12**, 862–870 (2011). 21865087 10.1016/S1470-2045(11)70213-3 PMC3172992

[28] Stanley, M. et al. Evidence for an HPV one-dose schedule. *Vaccine* **42**, S16–S21 (2024). 39521566 10.1016/j.vaccine.2024.01.046

[29] Leung, T. F. et al. Comparative immunogenicity and safety of human papillomavirus (HPV)-16/18 AS04-adjuvanted vaccine and 4vHPV vaccine administered according to two- or three-dose schedules in girls aged 9-14 years: Results to month 36 from a randomized trial. *Vaccine* **36**, 98–106 (2018). 29174109 10.1016/j.vaccine.2017.11.034

[30] Smolen, K. K. et al. Age of recipient and number of doses differentially impact human B and T cell immune memory responses to HPV vaccination. *Vaccine* **30**, 3572–3579 (2012). 22469863 10.1016/j.vaccine.2012.03.051

[31] Einstein, M. H. et al. Comparative humoral and cellular immunogenicity and safety of human papillomavirus (HPV)-16/18 AS04-adjuvanted vaccine and HPV-6/11/16/18 vaccine in healthy women aged 18-45 years: follow-up through Month 48 in a Phase III randomized study. *Hum. Vaccin Immunother.* **10**, 3455–3465 (2014). 25483700 10.4161/hv.36117 PMC4514093

[32] Watson-Jones, D. et al. Durability of immunogenicity at 5 years after a single dose of human papillomavirus vaccine compared with two doses in Tanzanian girls aged 9-14 years: results of the long-term extension of the DoRIS randomised trial. *Lancet Glob. Health* **13**, e319–e328 (2025). 39890232 10.1016/S2214-109X(24)00477-7 PMC11783036

[33] Bornstein, J. et al. Three-Year Follow-up of 2-Dose Versus 3-Dose HPV Vaccine. *Pediatrics.* **147**, e20194035 (2021). 33386332 10.1542/peds.2019-4035

[34] Klein, N. P. et al. Immunogenicity and Safety of Extended-Interval 2-Dose Regimens of 9vHPV Vaccine. *Pediatrics.* **154**, e2023064693 (2024). 38978512 10.1542/peds.2023-064693

[35] Bissett, S. L. et al. Seropositivity to non-vaccine incorporated genotypes induced by the bivalent and quadrivalent HPV vaccines: A systematic review and meta-analysis. *Vaccine* **35**, 3922–3929 (2017). 28633892 10.1016/j.vaccine.2017.06.028

[36] Cason, J. & Mant, C. A. High-risk mucosal human papillomavirus infections during infancy & childhood. *J. Clin. Virol.* **32**, S52–S58 (2005). 15753012 10.1016/j.jcv.2004.12.007

[37] Schiller, J. The Biological Rationale for a One Dose HPV Vaccine. *HPV World.* **26**.

[38] Amanna, I. J., Carlson, N. E. & Slifka, M. K. Duration of humoral immunity to common viral and vaccine antigens. *N. Engl. J. Med* **357**, 1903–1915 (2007). 17989383 10.1056/NEJMoa066092

[39] Scherer, E. M. et al. Analysis of Memory B-Cell Responses Reveals Suboptimal Dosing Schedule of a Licensed Vaccine. *J. Infect. Dis.* **217**, 572–580 (2018). 29186468 10.1093/infdis/jix566 PMC5853470

[40] Barrett, J. R. et al. Analyses of human vaccine-specific circulating and bone marrow-resident B cell populations reveal benefit of delayed vaccine booster dosing with blood-stage malaria antigens. *Front Immunol.* **14**, 1193079 (2023). 38299155 10.3389/fimmu.2023.1193079 PMC10827869

[41] Gray, P. et al. Lack of detectable HPV18 antibodies in 14% of quadrivalent vaccinees in a longitudinal cohort study. *NPJ Vaccines* **9**, 146 (2024). 39138224 10.1038/s41541-024-00941-w PMC11322158

[42] Bhatla, N. et al. Impact of age at vaccination and cervical HPV infection status on binding and neutralizing antibody titers at 10 years after receiving single or higher doses of quadrivalent HPV vaccine. *Hum. Vaccin Immunother.* **19**, 2289242 (2023). 38078840 10.1080/21645515.2023.2289242 PMC10760374

[43] Einstein, M. H. et al. Comparison of long-term immunogenicity and safety of human papillomavirus (HPV)-16/18 AS04-adjuvanted vaccine and HPV-6/11/16/18 vaccine in healthy women aged 18-45 years: end-of-study analysis of a Phase III randomized trial. *Hum. Vaccin Immunother.* **10**, 3435–3445 (2014). 25483701 10.4161/hv.36121 PMC4514070

[44] Godi, A. et al. Relationship between Humoral Immune Responses against HPV16, HPV18, HPV31 and HPV45 in 12-15 Year Old Girls Receiving Cervarix(R) or Gardasil(R) Vaccine. *PLoS One* **10**, e0140926 (2015). 26495976 10.1371/journal.pone.0140926 PMC4619723

[45] Kemp, T. J. et al. HPV16/18 L1 VLP vaccine induces cross-neutralizing antibodies that may mediate cross-protection. *Vaccine* **29**, 2011–2014 (2011). 21241731 10.1016/j.vaccine.2011.01.001 PMC3046309

[46] Moscicki, A. B. et al. Immune responses elicited by a fourth dose of the HPV-16/18 AS04-adjuvanted vaccine in previously vaccinated adult women. *Vaccine* **31**, 234–241 (2012). 23063422 10.1016/j.vaccine.2012.09.037

[47] Dauner, J. G. et al. Characterization of the HPV-specific memory B cell and systemic antibody responses in women receiving an unadjuvanted HPV16 L1 VLP vaccine. *Vaccine* **28**, 5407–5413 (2010). 20591543 10.1016/j.vaccine.2010.06.018 PMC2913111

[48] Cyster, J. G. & Allen, C. D. C. B Cell Responses: Cell Interaction Dynamics and Decisions. *Cell* **177**, 524–540 (2019). 31002794 10.1016/j.cell.2019.03.016 PMC6538279

[49] Oh, J. E. et al. Migrant memory B cells secrete luminal antibody in the vagina. *Nature* **571**, 122–126 (2019). 31189952 10.1038/s41586-019-1285-1 PMC6609483

[50] Hoes, J. et al. Review of long-term immunogenicity following HPV vaccination: Gaps in current knowledge. *Hum. Vaccin Immunother.* **18**, 1908059 (2022). 34033518 10.1080/21645515.2021.1908059 PMC8920133

[51] Crotty, S. et al. Cutting edge: long-term B cell memory in humans after smallpox vaccination. *J. Immunol.* **171**, 4969–4973 (2003). 14607890 10.4049/jimmunol.171.10.4969

[52] Porras, C. et al. HPV16/18 antibodies 16-years after single dose of bivalent HPV vaccination: Costa Rica HPV vaccine trial. *J. Natl Cancer Inst. Monogr.* **2024**, 329–336 (2024). 39529529 10.1093/jncimonographs/lgae032 PMC11555268

[53] Basu, P. et al. Vaccine efficacy against persistent human papillomavirus (HPV) 16/18 infection at 10 years after one, two, and three doses of quadrivalent HPV vaccine in girls in India: a multicentre, prospective, cohort study. *Lancet Oncol.* **22**, 1518–1529 (2021). 34634254 10.1016/S1470-2045(21)00453-8 PMC8560643

[54] Lacey, C. J. HPV vaccination in HIV infection. *Papillomavirus Res* **8**, 100174 (2019). 31252073 10.1016/j.pvr.2019.100174 PMC6603434

[55] Carter, J. J. et al. Term immune memory responses to human papillomavirus (HPV) vaccination following 2 versus 3 doses of HPV vaccine. *Vaccine* **50**, 126817 (2025). 39914257 10.1016/j.vaccine.2025.126817

[56] Barnabas, R. V. et al. Single-dose HPV vaccination efficacy among adolescent girls and young women in Kenya (the KEN SHE Study): study protocol for a randomized controlled trial. *Trials* **22**, 661 (2021). 34579786 10.1186/s13063-021-05608-8 PMC8475401

[57] Pinna, D. et al. Clonal dissection of the human memory B-cell repertoire following infection and vaccination. *Eur. J. Immunol.* **39**, 1260–1270 (2009). 19404981 10.1002/eji.200839129 PMC3864550

[58] Jahnmatz, M. et al. Optimization of a human IgG B-cell ELISpot assay for the analysis of vaccine-induced B-cell responses. *J. Immunol. Methods* **391**, 50–59 (2013). 23454005 10.1016/j.jim.2013.02.009

[59] Aldakak, L. et al. Sex difference in the immunogenicity of the quadrivalent Human Papilloma Virus vaccine: Systematic review and meta-analysis. *Vaccine* **39**, 1680–1686 (2021). 33637386 10.1016/j.vaccine.2021.02.022

[60] Giuliano, A. R. et al. Immunogenicity of the 9-valent human papillomavirus vaccine: Post hoc analysis from five phase 3 studies. *Hum. Vaccin Immunother.* **21**, 2425146 (2025). 39840832 10.1080/21645515.2024.2425146 PMC12934126

[61] Giannini, S. L. et al. Enhanced humoral and memory B cellular immunity using HPV16/18 L1 VLP vaccine formulated with the MPL/aluminium salt combination (AS04) compared to aluminium salt only. *Vaccine* **24**, 5937–5949 (2006). 16828940 10.1016/j.vaccine.2006.06.005

[62] Safaeian, M. et al. Durable antibody responses following one dose of the bivalent human papillomavirus L1 virus-like particle vaccine in the Costa Rica Vaccine Trial. *Cancer Prev. Res (Philos.)* **6**, 1242–1250 (2013). 10.1158/1940-6207.CAPR-13-0203 PMC7605443 24189371

