# Supplementary material for: Memory B-cells elicited by different HPV vaccine regimens in the DoRIS randomised controlled trial
Source: NPJ Vaccines. 2025 Nov 28;10:274. doi: 10.1038/s41541-025-01313-8 (PMC12753722; doi:10.1038/s41541-025-01313-8)
Supplement: Supplementary file 1 — Wiggins et al. memory B-cell DoRIS Supplementary Data [file 41541_2025_1313_MOESM1_ESM.pdf]

Supplementary Data

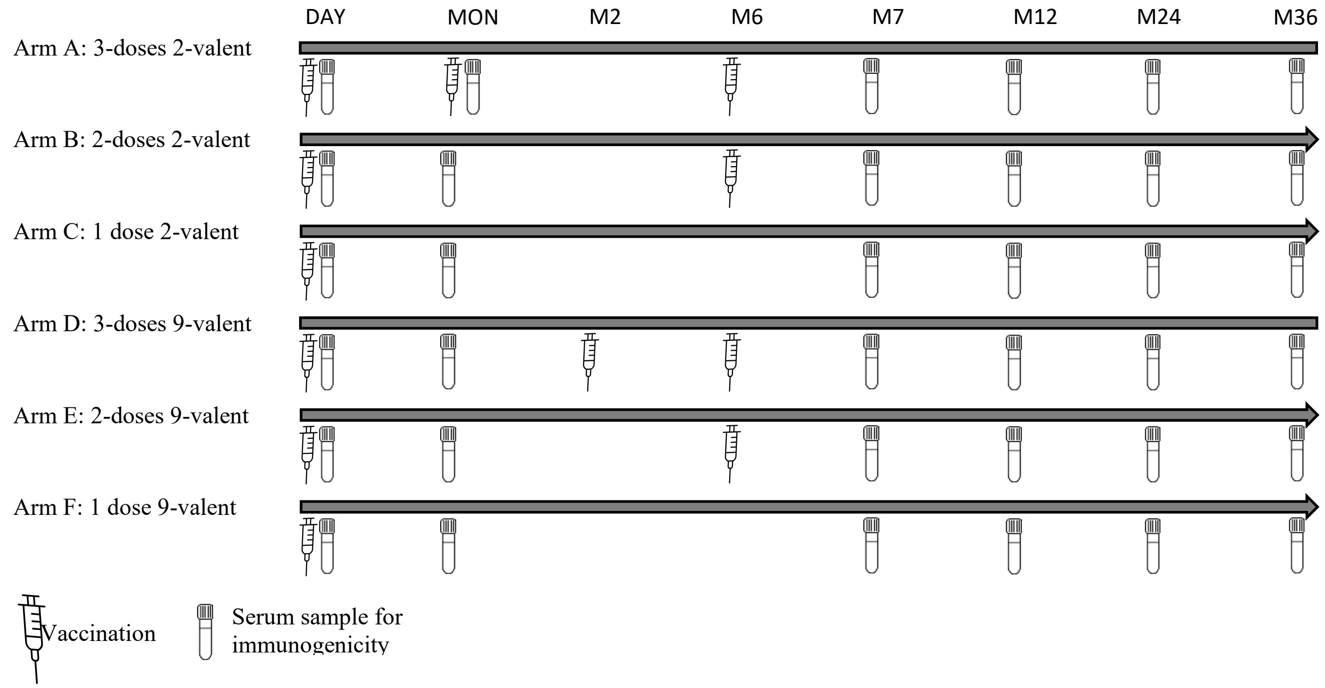

Supplementary Figure 1: Schematic of vaccination schedule for Cervarix® and Gardasil®9 and collection of samples for immunogencity (Baisley et al 2022)

Number of PBMC samples available for Memory B-cell ELISpot analysis

The 930 girls enrolled in the trial contributed 5522 peripheral blood mononuclear cell (PBMC) samples across the 6 study visits (Supplementary Table 1). Of the 5522 samples available across all timepoints, 5341 (96.7%) were included in the final analysis for the TVC. A small proportion (55, or <0.5%) had low viable cell counts (<75%) on thaw and were excluded from the analysis. Despite viability >75%, a further 126 samples had low total IgG (<100 total IgG positive spots in the first dilution (50,000 PBMCs)) following stimulation and were also excluded from the analysis.

Supplementary Table1: Sample availability for memory B cell ELISpots at each visit

	Visit						
	Day 0	Month 1	Month 7	Month 12	Month 24	Month 36	Total
Number providing samples	930	928	919	917	918	910	5522
Number (%) in TVC analysis[1]	879 (94.5%)	904 (97.4%)	908 (98.8%)	868 (94.7%)	892 (97.2%)	890 (97.8%)	5341
Number (%) in per-protocol analysis[2]							
HPV-16	813 (87.4%)	839 (90.4%)	847 (92.2%)	813 (88.7%)	831 (90.5%)	830 (91.2%)	4973
HPV-18	789 (84.8%)	814 (87.7%)	821 (89.3%)	788 (85.9%)	807 (87.9%)	806 (88.6%)	4825

[1]All DoRIS participants who received at least one dose of HPV vaccine, irrespective of their HPV DNA or serostatus at baseline. [2]DoRIS participants who were ELISA antibody negative and DNA negative at baseline (pre-vaccination) for the HPV genotype under analysis.

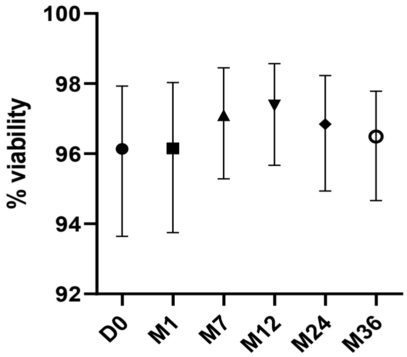

Number of values	930	928	919	918	920	910
Minimum	52.67	53.76	51.60	87.13	64.26	84.73
25% Percentile	93.65	93.75	95.28	95.67	94.94	94.66
Median	96.14	96.15	97.10	97.38	96.85	96.49
75% Percentile	97.94	98.03	98.45	98.57	98.23	97.78
Maximum	100.0	100.0	100.0	100.0	100.0	100.0
Range	47.33	46.24	48.40	12.87	35.74	15.27

Supplementary Figure 2: Percentage viability of isolated samples (TVC, after primary isolation N= 5,525). Graph shows medians and interquartile ranges

Median percentage viabilities following initial isolation of ~10mL heparinized whole blood were between 96.14 and 97.4, with the occasional sample viability dropping below 75% (n=15/5525 = 0.28% of all thawed samples). Samples with low viability on isolation were not included in the final analysis.

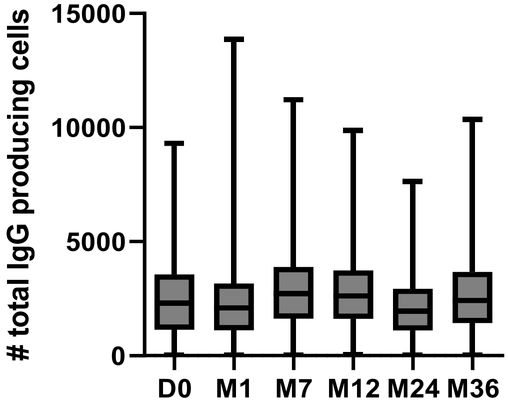

Dunn's multiple comparisons test	Mean rank diff.	Summary	Adjusted P Value
DAY 0 vs. Month 1	199.5	ns	0.0853
DAY 0 vs. Month 7	-364.5	****	<0.0001
DAY 0 vs.Month 12	-314.5	***	0.0002
DAY 0 vs. Month 24	311.2	***	0.0004
DAY 0 vs. Month 36	-156.3	ns	0.4680
Month 1 vs. Month 7	-564.0	****	<0.0001
Month 1 vs.Month 12	-514.0	****	<0.0001
Month 1 vs. Month 24	111.7	ns	>0.9999
Month 1 vs. Month 36	-355.8	****	<0.0001
Month 7 vs Month 12	49.97	ns	>0.9999
Month 7 vs. Month 24	675.7	****	<0.0001
Month 7 vs. Month 36	208.2	*	0.0492
Month 12 vs. Month 24	625.8	****	<0.0001
Month 12 vs. Month 36	158.3	ns	0.4055
Month 24 vs. Month 36	-467.5	****	<0.0001

Supplementary Figure.3: Total IgG-producing cells (raw data, not log-transformed) at each visit following stimulation with IL2 and R848 for 72 hours. Bars represent median and interquartile ranges

Median values for total IgG post-stimulation ranged from 1952 for Month 24 – 2719 for Month 7. There was a significant difference in the amount of total IgG producing cells following stimulation (Dunn's post-hoc multiple comparison test) between some timepoints, but no clear pattern emerged to indicate that the stimulation procedures or cellular responses at any particular timepoint were noticeably compromised or superior to any other timepoint. Months 7 and 12, which had overall the highest frequencies of antigen-specific memory B-cells also had the highest total IgG (2719, Month 7; 2622, Month 12).

Supplementary Table 2. Proportion (%) with detectable HPV-specific memory B-cells and $\log_{10}$ transformed geometric mean (GM) memory B-cell frequencies after 1, 2 or 3-doses of HPV vaccine in DoRIS trial (per protocol cohort[1])

	1 dose			2-doses			3-doses		
	N[1]	Positive[2] (%)	GM[3] (95% CI)	N[1]	Positive[2] (%)	GM[3] (95% CI)	N[1]	Positive[2] (%)	GM[3] (95% CI)
**Cervarix®**									
**Month 0**									
HPV-16	140	57 (40.7%)	0.006 (0.004 -0.009)	136	60 (44.1%)	0.006 (0.004 -0.009)	133	66 (49.6%)	0.009 (0.006 -0.013)
HPV-18	134	51 (38.1%)	0.005 (0.004 -0.007)	135	60 (44.4%)	0.006 (0.004 -0.009)	129	62 (48.1%)	0.007 (0.005 -0.010)
**Month 1**									
HPV-16	145	106 (73.1%)	0.030 (0.021 -0.044)	141	111 (78.7%)	0.039 (0.027 -0.056)	138	98 (71.0%)	0.025 (0.017 -0.036)
HPV-18	138	98 (71.0%)	0.025 (0.017 -0.036)	140	99 (70.7%)	0.026 (0.018 -0.037)	133	88 (66.2%)	0.019 (0.013 -0.029)
**Month 7**									
HPV-16	144	131 (91.0%)	0.074 (0.055 -0.101)	141	141 (100%)	0.588 (0.498 -0.694)	140	140 (100%)	0.769 (0.655 -0.903)
HPV-18	137	127 (92.7%)	0.063 (0.047 -0.085)	140	138 (98.6%)	0.359 (0.295 -0.438)	135	134 (99.3%)	0.428 (0.356 -0.516)
**Month 12**									
HPV-16	136	124 (91.2%)	0.078 (0.060 -0.103)	133	127 (95.5%)	0.130 (0.104 -0.164)	134	128 (95.5%)	0.183 (0.142 -0.236)
HPV-18	130	120 (92.3%)	0.074 (0.056 -0.096)	132	123 (93.2%)	0.098 (0.076 -0.126)	129	125 (96.9%)	0.148 (0.119 -0.185)
**Month 24**									
HPV-16	145	104 (71.7%)	0.036 (0.024 -0.053)	136	116 (85.3%)	0.103 (0.072 -0.147)	134	113 (84.3%)	0.114 (0.077 -0.167)
HPV-18	138	86 (62.3%)	0.023 (0.015 -0.036)	135	104 (77.0%)	0.051 (0.034 -0.075)	131	100 (76.3%)	0.065 (0.042 -0.100)
**Month 36**									
HPV-16	142	83 (58.5%)	0.013 (0.009 -0.018)	140	87 (62.1%)	0.016 (0.011 -0.024)	135	101 (74.8%)	0.034 (0.023 -0.050)
HPV-18	136	77 (56.6%)	0.011 (0.008 -0.017)	139	80 (57.6%)	0.013 (0.009 -0.019)	130	87 (66.9%)	0.023 (0.015 -0.034)
**Gardasil®9®**									
**Month 0**									
HPV-16	138	63 (45.7%)	0.007 (0.005 -0.010)	135	52 (38.5%)	0.005 (0.004 -0.008)	131	61 (46.6%)	0.008 (0.005 -0.011)
HPV-18	129	51 (39.5%)	0.005 (0.004 -0.007)	130	50 (38.5%)	0.005 (0.004 -0.008)	132	52 (39.4%)	0.006 (0.004 -0.008)
**Month 1**									
HPV-16	142	115 (81.0%)	0.045 (0.032 -0.063)	137	108 (78.8%)	0.039 (0.027 -0.055)	136	114 (83.8%)	0.054 (0.039 -0.075)
HPV-18	133	91 (68.4%)	0.019 (0.013 -0.027)	133	88 (66.2%)	0.016 (0.011 -0.023)	137	99 (72.3%)	0.025 (0.018 -0.037)
**Month 7**									
HPV-16	142	129 (90.8%)	0.080 (0.059 -0.109)	139	139 (100%)	0.897 (0.774 -1.039)	141	141 (100%)	0.758 (0.648 -0.886)
HPV-18	133	120 (90.2%)	0.050 (0.037 -0.068)	134	132 (98.5%)	0.324 (0.265 -0.397)	142	139 (97.9%)	0.285 (0.227 -0.356)
**Month 12**									
HPV-16	142	132 (93.0%)	0.111 (0.086 -0.143)	135	131 (97.0%)	0.185 (0.149 -0.231)	133	131 (98.5%)	0.238 (0.201 -0.281)
HPV-18	134	122 (91.0%)	0.074 (0.056 -0.096)	130	125 (96.2%)	0.110 (0.088 -0.138)	133	128 (96.2%)	0.132 (0.106 -0.164)
**Month 24**									
HPV-16	143	105 (73.4%)	0.049 (0.032 -0.074)	137	114 (83.2%)	0.113 (0.076 -0.167)	136	117 (86.0%)	0.144 (0.099 -0.209)
HPV-18	134	81 (60.4%)	0.020 (0.013 -0.032)	132	93 (70.5%)	0.045 (0.029 -0.070)	137	105 (76.6%)	0.067 (0.044 -0.102)
**Month 36**									
HPV-16	137	81 (59.1%)	0.014 (0.010 -0.022)	138	97 (70.3%)	0.026 (0.018 -0.038)	138	104 (75.4%)	0.040 (0.027 -0.058)
HPV-18	129	66 (51.2%)	0.010 (0.006 -0.014)	133	78 (58.6%)	0.014 (0.009 -0.021)	139	91 (65.5%)	0.024 (0.016 -0.036)

[1]DoRIS participants who were ELISA antibody negative and DNA negative at baseline (pre-vaccination) for the HPV genotype under analysis. [2]Number (%) with detectable HPV-specific memory B-cells. [3]Geometric mean $\log_{10}$ transformed % of HPV-specific circulating memory B-cells. Values below the assay limit of quantitation (LLQ) are set to 0.5*LLQ for analysis. Supplementary Table 1 is referred to in the main text Results section and informs Fig. 1.

Supplementary Table 3. Geometric mean (GM) B-cell responses after 1, 2 or 3-doses of HPV vaccine in DoRIS trial (total vaccinated cohort[1])

	1 dose		2-doses		3-doses		Geometric mean memory B-cell ratio (95% CI)		
	**N (%)[2]**	**GM[3] (95% CI)**	**N (%)[2]**	**GM[3] (95% CI)**	**N (%)[2]**	**GM[3] (95% CI)**	**1 dose / 2-dose**	**1 dose / 3-dose**	**2-dose / 3-dose**
**Cervarix®**									
**Month 1**									
	N=151		***N=152***		***N=152***				
HPV-16	111 (73.5%)	0.031 (0.021 -0.044)	120 (78.9%)	0.039 (0.028 -0.055)	111 (73.0%)	0.028 (0.020 -0.039)	0.79 (0.48 -1.29)	1.11 (0.68 -1.81)	1.41 (0.87 -2.30)
HPV-18	104 (68.9%)	0.022 (0.015 -0.032)	107 (70.4%)	0.025 (0.017 -0.036)	104 (68.4%)	0.022 (0.015 -0.031)	0.88 (0.53 -1.47)	1.03 (0.62 -1.70)	1.16 (0.70 -1.93)
**Month 7**									
	N=150		***N=151***		***N=153***				
HPV-16	136 (90.7%)	0.074 (0.055 -0.099)	151 (100.0%)	0.591 (0.502 -0.696)	152 (99.3%)	0.703 (0.588 -0.841)	0.12 (0.09 -0.17)	0.10 (0.08 -0.14)	0.84 (0.62 -1.14)
HPV-18	139 (92.7%)	0.062 (0.047 -0.081)	149 (98.7%)	0.363 (0.301 -0.438)	152 (99.3%)	0.424 (0.355 -0.505)	0.17 (0.12 -0.23)	0.15 (0.11 -0.20)	0.86 (0.63 -1.16)
**Month 12**									
	N=141		***N=143***		***N=147***				
HPV-16	129 (91.5%)	0.078 (0.060 -0.101)	137 (95.8%)	0.132 (0.106 -0.164)	141 (95.9%)	0.175 (0.139 -0.222)	0.59 (0.42 -0.82)	0.44 (0.32 -0.62)	0.75 (0.54 -1.05)
HPV-18	129 (91.5%)	0.069 (0.053 -0.091)	134 (93.7%)	0.097 (0.076 -0.123)	142 (96.6%)	0.143 (0.116 -0.176)	0.71 (0.51 -1.00)	0.49 (0.35 -0.68)	0.68 (0.49 -0.95)
**Month 24**									
	N=151		***N=146***		***N=147***				
HPV-16	108 (71.5%)	0.035 (0.024 -0.053)	121 (82.9%)	0.090 (0.063 -0.129)	126 (85.7%)	0.117 (0.082 -0.167)	0.39 (0.23 -0.66)	0.30 (0.18 -0.51)	0.77 (0.45 -1.30)
HPV-18	94 (62.3%)	0.023 (0.015 -0.035)	112 (76.7%)	0.050 (0.034 -0.073)	111 (75.5%)	0.061 (0.040 -0.092)	0.46 (0.26 -0.81)	0.38 (0.21 -0.66)	0.82 (0.46 -1.45)
**Month 36**									
	N=148		***N=150***		***N=148***				
HPV-16	86 (58.1%)	0.012 (0.009 -0.018)	96 (64.0%)	0.017 (0.012 -0.025)	112 (75.7%)	0.037 (0.026 -0.053)	0.71 (0.42 -1.18)	0.33 (0.20 -0.56)	0.47 (0.28 -0.79)
HPV-18	83 (56.1%)	0.011 (0.008 -0.017)	87 (58.0%)	0.013 (0.009 -0.020)	99 (66.9%)	0.023 (0.016 -0.034)	0.85 (0.50 -1.45)	0.49 (0.29 -0.83)	0.58 (0.34 -0.98)
**Gardasil®9®**									
**Month 1**									
	N=149		***N=150***		***N=150***				
HPV-16	119 (79.9%)	0.043 (0.031 -0.060)	119 (79.3%)	0.039 (0.028 -0.05)	125 (83.3%)	0.053 (0.039 -0.073)	1.11 (0.70 -1.77)	0.81 (0.51 -1.28)	0.72 (0.46 -1.15)
HPV-18	101 (67.8%)	0.018 (0.013 -0.025)	96 (64.0%)	0.015 (0.010 -0.021)	106 (70.7%)	0.023 (0.016 -0.033)	1.21 (0.74 -1.99)	0.77 (0.47 -1.26)	0.63 (0.39 -1.04)
**Month 7**									
	N=149		***N=151***		***N=154***				
HPV-16	136 (91.3%)	0.080 (0.060 -0.108)	151 (100.0%)	0.902 (0.785 -1.036)	154 (100.0%)	0.732 (0.628 -0.853)	0.09 (0.07 -0.12)	0.11 (0.08 -0.15)	1.23 (0.92 -1.65)
HPV-18	133 (89.3%)	0.047 (0.035 -0.063)	149 (98.7%)	0.342 (0.283 -0.413)	151 (98.1%)	0.288 (0.234 -0.355)	0.14 (0.10 -0.19)	0.16 (0.12 -0.23)	1.19 (0.86 -1.65)
**Month 12**									
	N=147		N=145		N=145				
HPV-16	136 (92.5%)	0.106 (0.082 -0.137)	141 (97.2%)	0.182 (0.148 -0.225)	142 (97.9%)	0.230 (0.193 -0.273)	0.58 (0.43 -0.79)	0.46 (0.34 -0.63)	0.79 (0.59 -1.07)

HPV-18	135 (91.8%)	0.076 (0.059 -0.097)	139 (95.9%)	0.108 (0.087 -0.133)	140 (96.6%)	0.134 (0.109 -0.164)	0.70 (0.52 -0.96)	0.57 (0.41 -0.77)	0.80 (0.59 -1.10)
**Month 24**									
		N=150		***N=149***		***N=149***			
HPV-16	110 (73.3%)	0.048 (0.032 -0.071)	123 (82.6%)	0.106 (0.072 -0.155)	130 (87.2%)	0.153 (0.108 -0.216)	0.45 (0.27 -0.76)	0.31 (0.18 -0.53)	0.69 (0.41 -1.17)
HPV-18	93 (62.0%)	0.021 (0.014 -0.032)	103 (69.1%)	0.040 (0.027 -0.062)	112 (75.2%)	0.062 (0.042 -0.093)	0.53 (0.30 -0.94)	0.34 (0.19 -0.61)	0.65 (0.36 -1.16)
**Month 36**									
		N=143		***N=150***		***N=151***			
HPV-16	84 (58.7%)	0.014 (0.010 -0.021)	106 (70.7%)	0.027 (0.018 -0.039)	115 (76.2%)	0.042 (0.029 -0.061)	0.54 (0.32 -0.92)	0.34 (0.20 -0.58)	0.63 (0.37 -1.06)
HPV-18	73 (51.0%)	0.010 (0.006 -0.014)	90 (60.0%)	0.015 (0.010 -0.022)	101 (66.9%)	0.025 (0.017 -0.036)	0.64 (0.37 -1.11)	0.39 (0.23 -0.67)	0.60 (0.35 -1.03)

[1]DoRIS participants who received at least one dose of vaccine, irrespective of their HPV DNA or serostatus at baseline. [2]Number with detectable HPV-specific memory B-cells at each timepoint. [3]Geometric mean % of HPV-specific circulating memory B-cells. Values below the assay limit of quantitation (LLQ) are set to 0.5*LLQ for analysis.

Number of antigen-specific spots per 1x10^5 IgG-producing cells (TVC)

At Day 0, the median number of spots was 0, although there were spots above background (combined media only wells + KLH wells) present in approximately 45% of samples (range 0 – 20 spots, Cervarix®; 0 – 25 spots, Gardasil®9). These values were independent of HPV16 and/or 18 positivity at baseline.

Of the 58 girls who were HPV16 seropositive or DNA positive at baseline, 23 (39.7%) had detectable HPV16-specific memory B cells at baseline vs 363/821 (44.2%) of those who were HPV16 antibody negative and DNA negative (p=0.50). Geometric mean HPV16-specific memory B cell frequencies at baseline were 0.005 in those who were HPV16 seropositive or DNA positive vs 0.007 in those who were antibody/DNA negative (p=0.42). Of the 82 girls who were HPV18 seropositive or DNA positive at baseline, 38 (46.3%) had detectable HPV18-specific memory B cells at baseline vs 331/797 (41.5%) of those who were HPV18 antibody negative and DNA negative (p=0.40). Geometric mean HPV18-specific memory B cell frequencies at baseline were 0.007 in those who HPV18 seropositive or DNA positive vs 0.006 in those who were antibody/DNA negative (p=0.31).

Median HPV-specific spot counts were highest for HPV16 for both vaccines (Supplementary Fig. 4) at all timepoints post-vaccination. The greatest number of spots were present at Month 7 overall (median 3-dose Cervarix® HPV16 = 21, HPV18 = 11; 3-dose Gardasil®9 HPV16 = 19.5, HPV18 = 8) and numbers fell progressively to Month 36 (median spot count Month 12 3-dose Cervarix®, HPV16 = 6, HPV 18 =4; Month 24 3-dose Cervarix®, HPV16 = 4, HPV 18 =3; Month 36 3-dose Cervarix®, HPV16 = 2, HPV18 = 1).

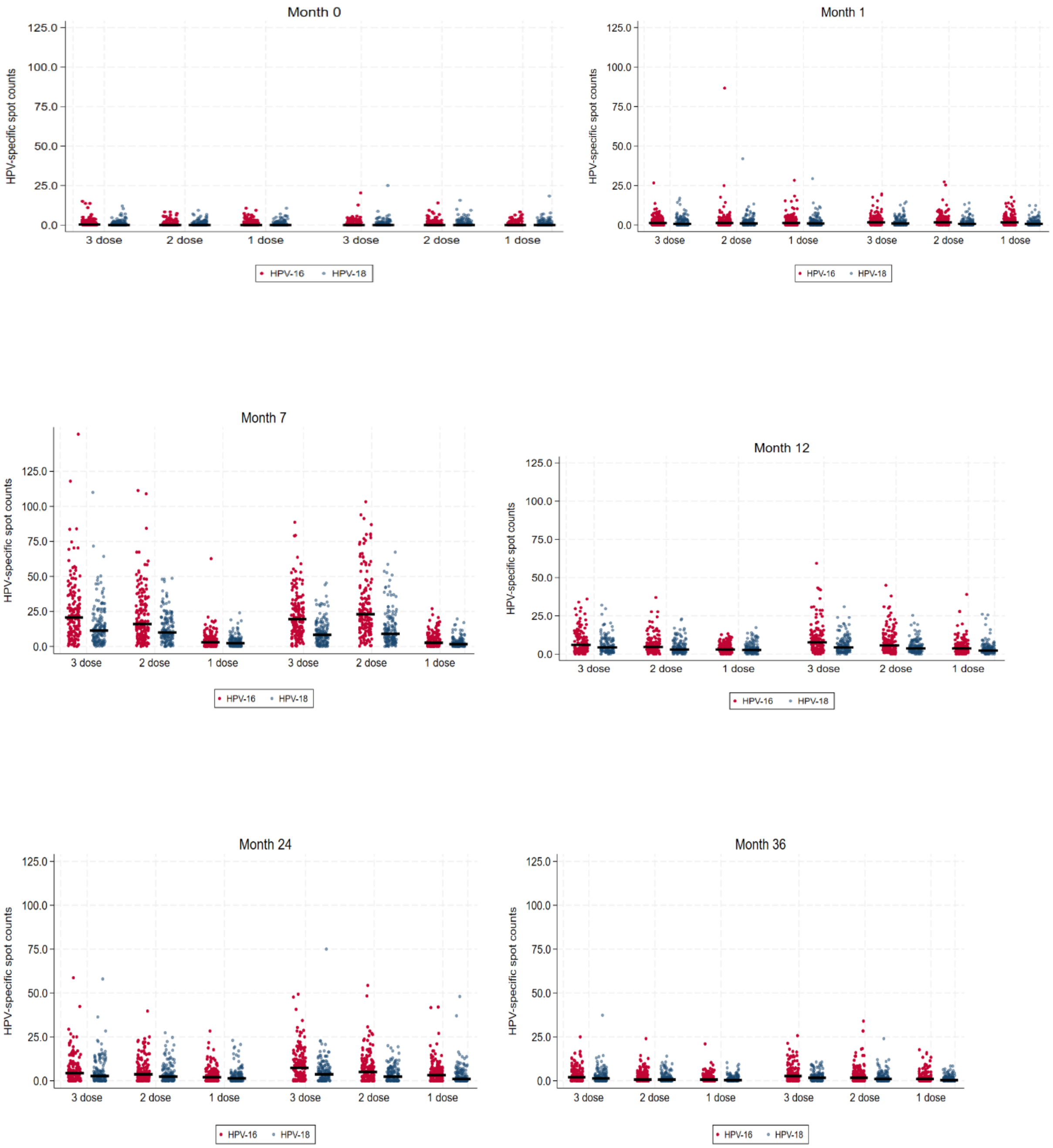

Supplementary Figure 4. <u>Number of HPV-specific IgG-producing cells at Day 0 and Months 1, 7, 12, 24 and 36 by arm</u> (total vaccinated cohort). Each data point represents a single individual and the line through the data points represents the median

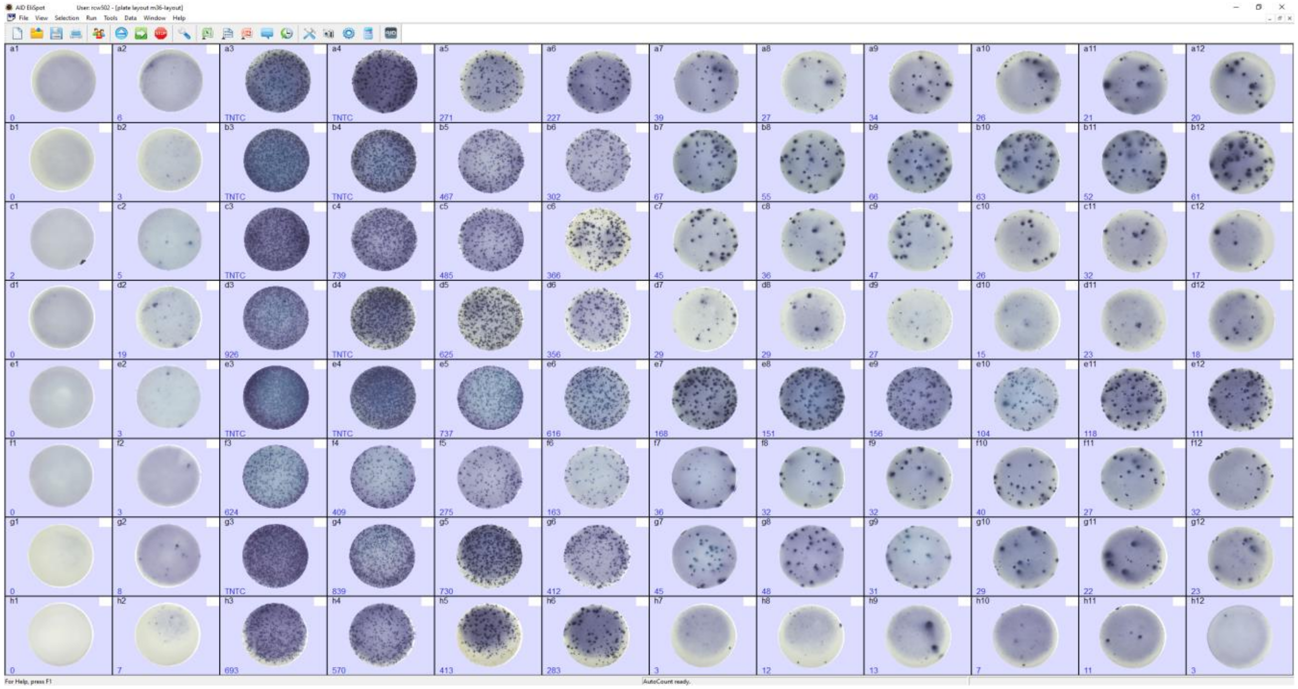

Supplementary Figure 5: Readout (IgG, total and antigen-specific) from AID ELISpot reader (column 1, media control; column 2, KLH control; columns 3-6; total IgG doubling dilutions starting concentration = 50,000 PBMCs; columns 7-9, antigen-specific spots from 100,000 PBMCs, HPV16; columns 10-12, antigen-specific spots from 100,000 PBMCs, HPV18). Each row represents one sample (all samples from the Month 7 timepoint).

Supplementary Table 4: Proportion with detectable HPV-specific memory B cells and geometric mean (GM) memory B cell frequencies after 1, 2 or 3-doses of HPV vaccine in DoRIS trial, among participants with undetectable HPV-specific memory B cells at baseline

	1 dose			2-doses			3-doses		
	N[1]	Positive[2] (%)	GM[3] (95% CI)	N[1]	Positive[2] (%)	GM[3] (95% CI)	N[1]	Positive[2] (%)	GM[3] (95% CI)
**Cervarix®®**									
**Month 0**									
HPV-16	87	0	–	85	0	–	71	0	–
HPV-18	89	0	–	83	0	–	77	0	–
**Month 1**									
HPV-16	84	60 (71.4%)	0.029 (0.018 -0.047)	82	64 (78.0%)	0.035 (0.022 -0.056)	70	50 (71.4%)	0.027 (0.016 -0.045)
HPV-18	86	62 (72.1%)	0.027 (0.017 -0.044)	81	62 (76.5%)	0.032 (0.020 -0.051)	76	53 (69.7%)	0.022 (0.013 -0.037)
**Month 7**									
HPV-16	83	74 (89.2%)	0.072 (0.048 -0.108)	83	83 (100.0%)	0.639 (0.507 -0.805)	70	69 (98.6%)	0.709 (0.530 -0.950)
HPV-18	86	79 (91.9%)	0.066 (0.045 -0.097)	81	80 (98.8%)	0.352 (0.270 -0.458)	76	76 (100.0%)	0.484 (0.381 -0.616)
**Month 12**									
HPV-16	80	74 (92.5%)	0.083 (0.059 -0.116)	79	75 (94.9%)	0.117 (0.086 -0.159)	70	67 (95.7%)	0.206 (0.146 -0.291)
HPV-18	82	75 (91.5%)	0.069 (0.049 -0.099)	79	72 (91.1%)	0.079 (0.056 -0.112)	75	74 (98.7%)	0.171 (0.131 -0.222)
**Month 24**									
HPV-16	85	62 (72.9%)	0.042 (0.024 -0.071)	79	65 (82.3%)	0.088 (0.054 -0.145)	68	61 (89.7%)	0.152 (0.094 -0.248)
HPV-18	87	54 (62.1%)	0.024 (0.013 -0.042)	78	59 (75.6%)	0.044 (0.026 -0.075)	72	57 (79.2%)	0.081 (0.045 -0.146)
**Month 36**									
HPV-16	83	49 (59.0%)	0.014 (0.008 -0.023)	83	56 (67.5%)	0.019 (0.012 -0.031)	71	55 (77.5%)	0.036 (0.022 -0.060)
HPV-18	84	49 (58.3%)	0.013 (0.008 -0.022)	82	46 (56.1%)	0.013 (0.008 -0.022)	76	54 (71.1%)	0.027 (0.016 -0.045)
**Gardasil-9®**									
**Month 0**									
HPV-16	80	0	–	91	0	–	79	0	–
HPV-18	86	0	–	89	0	–	86	0	–
**Month 1**									
HPV-16	78	62 (79.5%)	0.041 (0.026 -0.066)	88	68 (77.3%)	0.036 (0.023 -0.057)	77	64 (83.1%)	0.047 (0.030 -0.072)
HPV-18	84	61 (72.6%)	0.021 (0.013 -0.033)	86	55 (64.0%)	0.017 (0.010 -0.027)	83	58 (69.9%)	0.020 (0.012 -0.032)
**Month 7**									
HPV-16	77	70 (90.9%)	0.075 (0.050 -0.112)	89	89 (100%)	0.910 (0.763 -1.086)	78	78 (100%)	0.689 (0.543 -0.876)
HPV-18	83	76 (91.6%)	0.051 (0.035 -0.074)	86	85 (98.8%)	0.331 (0.260 -0.422)	86	84 (97.7%)	0.266 (0.198 -0.357)
**Month 12**									
HPV-16	76	68 (89.5%)	0.094 (0.063 -0.141)	86	82 (95.3%)	0.154 (0.113 -0.210)	74	72 (97.3%)	0.228 (0.176 -0.296)

HPV-18	80	72 (90.0%)	0.067 (0.046 -0.097)	85	81 (95.3%)	0.107 (0.081 -0.142)	82	77 (93.9%)	0.107 (0.078 -0.147)
**Month 24**									
HPV-16	78	57 (73.1%)	0.053 (0.030 -0.094)	90	76 (84.4%)	0.133 (0.082 -0.216)	76	66 (86.8%)	0.144 (0.089 -0.234)
HPV-18	84	52 (61.9%)	0.021 (0.012 -0.037)	86	62 (72.1%)	0.049 (0.028 -0.085)	85	62 (72.9%)	0.057 (0.033 -0.100)
**Month 36**									
HPV-16	74	45 (60.8%)	0.017 (0.010 -0.031)	89	60 (67.4%)	0.023 (0.014 -0.038)	76	59 (77.6%)	0.045 (0.027 -0.075)
HPV-18	79	46 (58.2%)	0.013 (0.008 -0.023)	87	50 (57.5%)	0.013 (0.008 -0.022)	84	59 (70.2%)	0.032 (0.019 -0.053)

[1]DoRIS participants who had no detectable memory B cells at baseline for the HPV genotype under analysis. [2]Number (%) with any detectable HPV-specific memory B cells. [3]Geometric mean HPV-specific memory B cell frequency. Values below the assay limit of quantitation (LLQ) are set to 0.5*LLQ for analysis.

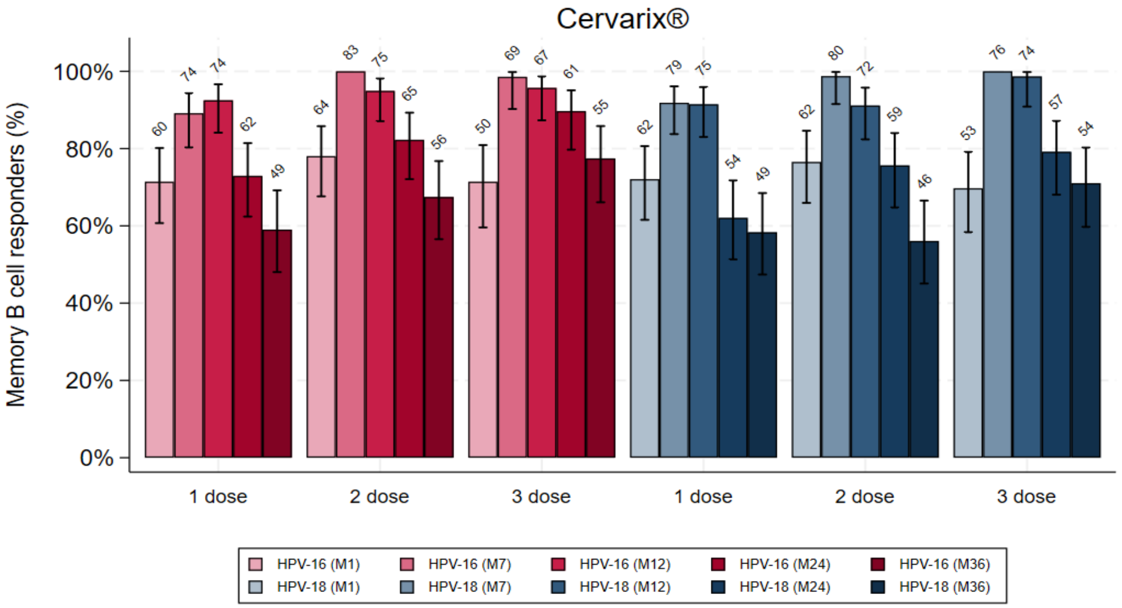

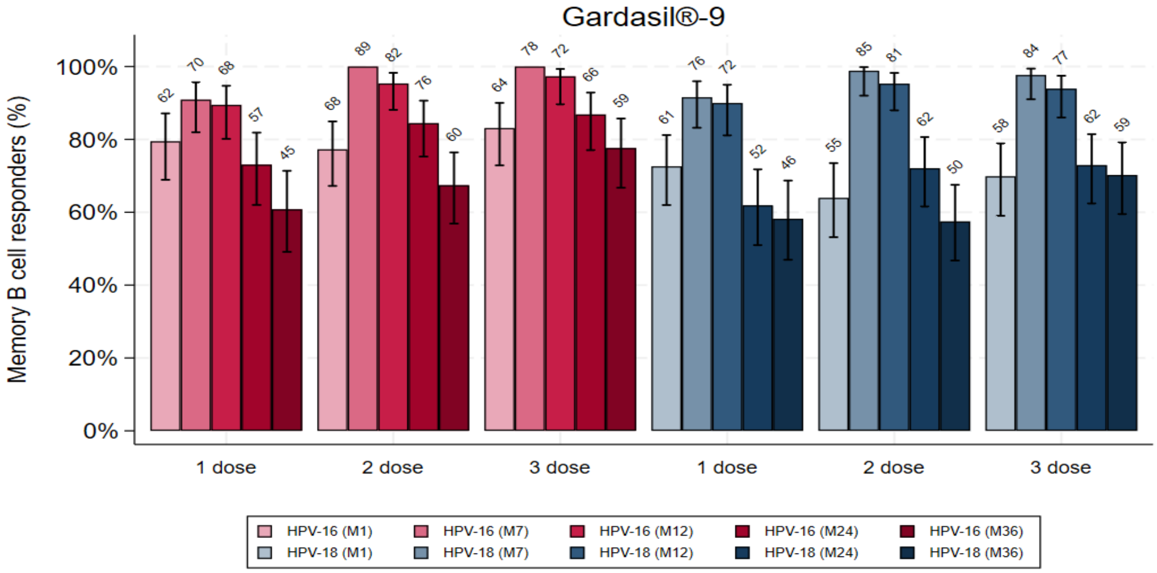

Supplementary Figure 6: Proportion with detectable HPV-specific B cell responses over time among participants with undetectable HPV-specific memory B cells at baseline. Bars represent 95% CI, and numbers are N with detectable responses

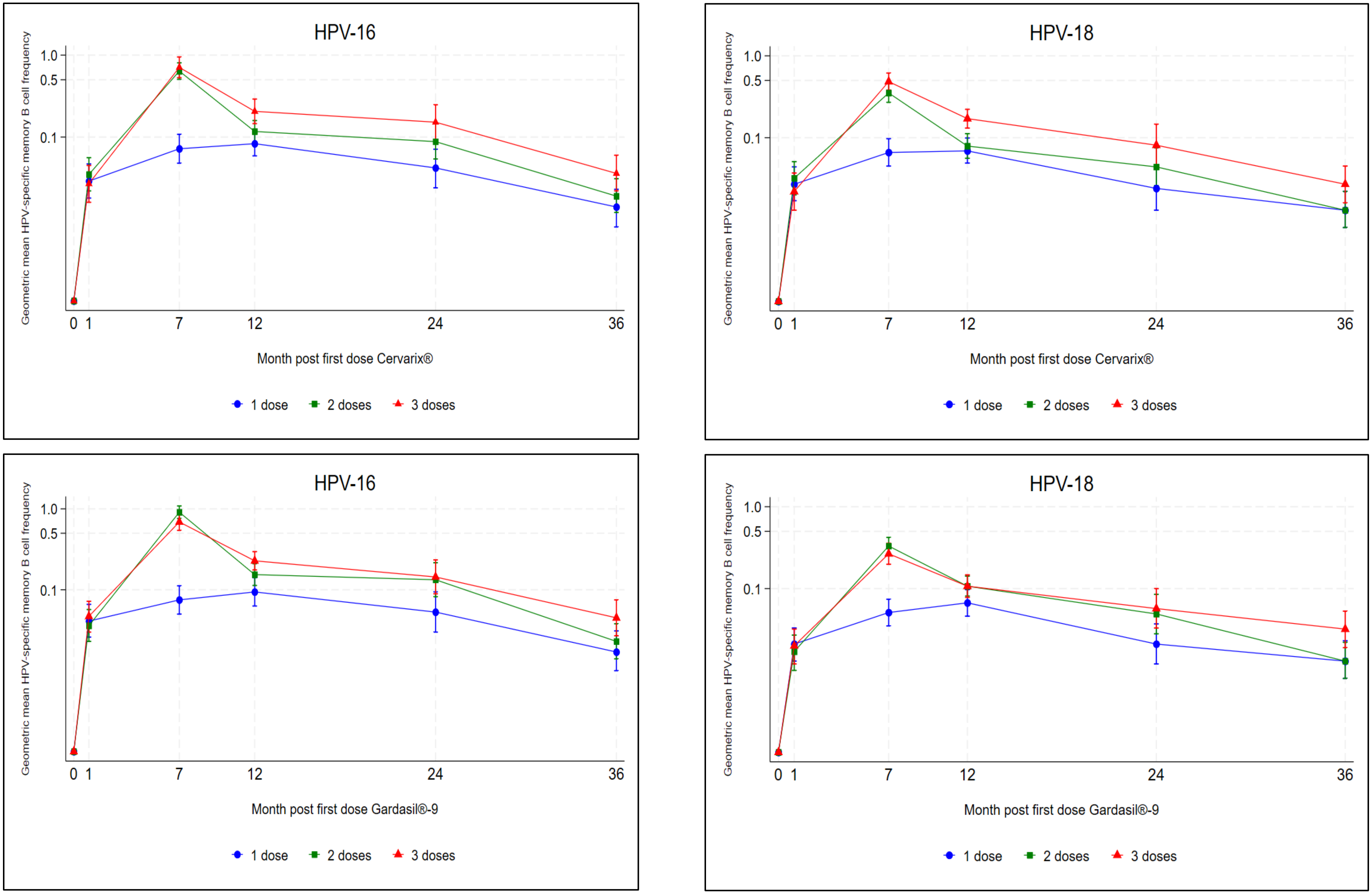

Supplementary Figure 7: Geometric mean HPV16 (left) and HPV18 (right) specific memory B cell frequency by number of doses of Cervarix® (top) and Gardasil-9 (bottom) and study visit among participants with undetectable HPV-specific memory B cells at baseline

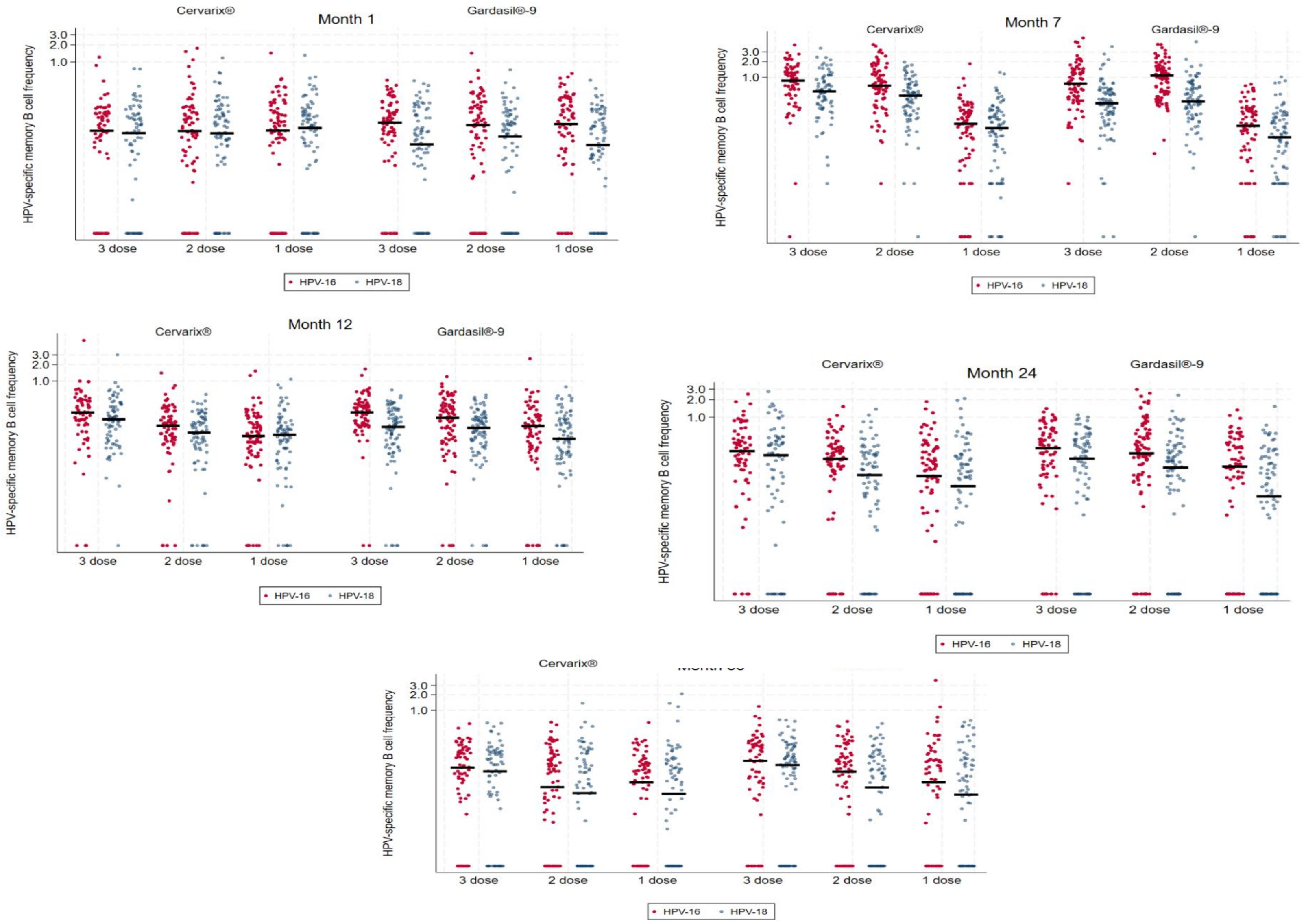

Supplementary Figure 8: <u>Distribution of HPV-specific memory B cell responses at months 1, 7, 12 and 24, by arm among participants with undetectable HPV-specific memory B cells at baseline. Each data point represents a single individual and the line through the data points represents the median</u>
